# GLA:D^®^ Back group-based patient education integrated with exercises to support self-management of back pain - development, theories and scientific evidence -

**DOI:** 10.1186/s12891-018-2334-x

**Published:** 2018-11-29

**Authors:** Per Kjaer, Alice Kongsted, Inge Ris, Allan Abbott, Charlotte Diana Nørregaard Rasmussen, Ewa M. Roos, Søren T. Skou, Tonny Elmose Andersen, Jan Hartvigsen

**Affiliations:** 1 Department of Sports Science and Clinical Biomechanics, University of Southern Denmark, Campusvej 55, 5230 Odense M, Denmark; 2 Department of Applied Health Services, University College Lillebaelt, Niels Bohrs Alle 1, 5230 Odense M, Denmark; 3 Nordic Institute of Chiropractic and Clinical Biomechanics, Campusvej 55, 5230 Odense M, Denmark; 4 Department of Medical and Health Sciences, Division of Physiotherapy, Faculty of Health Sciences, Sandbäcksgatan 7/3, University Hospital Campus, Linköping University, 581 83 Linköping, Sweden; 5 National Research Centre for the Working Environment, Lersø Parkallé 105, 2100 Copenhagen Ø, Denmark; 6 Research Unit for Musculoskeletal Function and Physiotherapy, Department of Sports Science and Clinical Biomechanics, University of Southern Denmark, Campusvej 55, 5230 Odense M, Denmark; 7 Department of Physiotherapy and Occupational Therapy, Næstved-Slagelse-Ringsted Hospitals, Region Zealand, 4200 Slagelse, Denmark; 8 Department of Psychology, University of Southern Denmark, Campusvej 55, 5230 Odense M, Denmark

**Keywords:** Back pain, Patient education, Exercise therapy, Intervention development, Primary health care

## Abstract

**Background:**

Clinical guidelines recommend that people with back pain be given information and education about their back pain, advice to remain active and at work, and exercises to improve mobility and physical activity. Guidelines, however, rarely describe how this is best delivered. The aim of this paper is to present the development, theories, and underlying evidence for ‘GLA:D Back’ - a group education and exercise program that translates guideline recommendations into a clinician-delivered program for the promotion of self-management in people with persistent/recurrent back pain.

**Methods:**

GLA:D Back, which included a rationale and objectives for the program, theory and evidence for the interventions, and program materials, was developed using an iterative process. The content of patient education and exercise programs tested in randomised trials was extracted and a multidisciplinary team of expert researchers and clinicians prioritised common elements hypothesised to improve back pain beliefs and management skills. The program was tested on eight people with persistent back pain in a university clinic and 152 patients from nine primary care physiotherapy and chiropractic clinics. Following feedback from the clinicians and patients involved, the working version of the program was created.

**Results:**

Educational components included *pain mechanisms*, *pain modulation*, *active coping strategies*, *imaging*, *physical activity*, and *exercise* that emphasised a balance between the sum of demands and the individual’s capacity*.* These were operationalised in PowerPoint presentations with supporting text to aid clinicians in delivering two one-hour patient education lectures.

The exercise program included 16 supervised one-hour sessions over 8 weeks, each comprising a warm-up section and eight types of exercises for general flexibility and strengthening of six different muscle groups at four levels of difficulty. The aims of the exercises were to improve overall back fitness and, at the same time, encourage patients to explore variations in movement by incorporating education content into the exercise sessions.

**Conclusion:**

From current best evidence about prognostic factors in back pain and effective treatments for back pain, research and clinical experts developed a ready-to-use structured program - GLA:D® Back - to support self-management for people with persistent/recurrent back pain.

**Electronic supplementary material:**

The online version of this article (10.1186/s12891-018-2334-x) contains supplementary material, which is available to authorized users.

## Background

Evidence-based clinical practice guidelines universally recommend patient education, advice to remain active and at work, and exercises as frontline interventions to help people with persistent and/or recurrent episodes of back pain to self-manage [1]. In spite of this, non-evidence-based practices including excessive testing and imaging, prescription of opioids, spinal injections and surgery are commonly used for these patients, and a significant evidence-practice gap exists [2]. Leading back pain researchers are calling for implementation of guideline recommendations in order to avoid harmful treatments in all settings globally [3].

An example of a successful implementation of clinical guideline recommendations is the GLA:D (Good Life with osteoArthritis in Denmark) for the knee and hip [4]. This program consists of a two-day course that trains clinicians in delivering GLA:D, a standardised evidence-based program for knee and hip pain. GLA:D includes two sessions of patient education and 12 sessions of supervised exercise therapy aimed at teaching patients to self-manage their pain and functional limitations, as well as registration of their data in a clinical database, where they are monitored for one year on a number of outcomes such as pain, physical function, pain medication and quality of life [5]. Since 2013, more than 1000 physiotherapists have been certified and around 36,000 patients included in the clinical registry in Denmark [4, 6]. After participating in GLA:D knee and hip, pain decreased by 26–27%, function improved, fewer people took pain-killers and fewer people were on sick leave [6]. In addition, GLA:D is currently being implemented in Canada, Australia, and China [6]. The GLA:D approach appears to be an effective, feasible and fast method to implement recommendations from clinical guidelines in clinical practice, and a similar approach might be useful to implement recommendations from clinical guidelines for back pain.

Due to the successful implementation of the GLAD knee and hip program, we had requests from clinicians for a similar program for people with back pain. From our networks and collaboration with clinicians, we also had very positive responses to our initial ideas and we therefore found it timely to develop GLA:D Back.

The overall aim of this project was to develop and implement GLA:D Back, an intervention that compiles elements of effective and generally recommended interventions into a standardised care package that is feasible to be delivered by clinicians in primary care (Kongsted A, Ris I, Kjaer P, Vach W, Morso L, Hartvigsen J: GLA:D® Back: Implementation of group-based patient education integrated with exercises to support self-management of back pain. Protocol for a hybrid effectiveness-implementation study, submitted). The intention was to promote self-management for people with persistent or recurrent back pain. A self-management intervention has previously been defined as ‘a structured, taught, or self-taught course with distinct components principally aimed at patients (rather than carers) with the goal of improving the participants’ health status or quality of life by teaching them skills to apply to everyday situations’ [7]. The following components have been suggested: psychological (including behavioural or cognitive therapy), mind-body therapies (including relaxation, meditation, or guided imagery), physical activity (including any form of exercise), lifestyle (such as dietary advice and sleep management), and pain education (such as understanding the condition and how to take medication effectively).

In this paper, we present the development, theories, and underlying scientific evidence for the GLA:D Back program, which consists of a standardised clinician-delivered care program for back pain comprising group education and supervised exercises aimed at supporting self-management in people seeking care due to persistent and/or recurrent back pain.

## Methods

In Section 1, we describe the *rationale* for developing GLA:D Back, in Section 2, we describe the *objectives* of the intervention and the hypothesised model of change, Section 3 describes the *program design* and the underlying *theories and evidence*, and Section 4, the *final content* of the program. The implementation and the evaluation of the intervention are described in a separate protocol paper that describes the educational intervention targeted at the clinicians, who will deliver GLA:D Back (Kongsted A, Ris I, Kjaer P, Vach W, Morso L, Hartvigsen J: GLA:D® Back: Implementation of group-based patient education integrated with exercises to support self-management of back pain. Protocol for a hybrid effectiveness-implementation study, submitted).

The reporting of the intervention development is inspired by the framework of Intervention Mapping, which is a method for developing behavioural change interventions [8–10]. It is particularly useful in complex intervention development as a theoretical framework for optimising potential effects of a new intervention [11]. Accordingly, Section 1 is primarily based on literature reviews and extraction of themes relevant for a group-delivered intervention focusing on self-management. Group and consensus discussions led to the outlining of the objectives for the intervention in Section 2. Section 3 is based on literature reviews of the theory and evidence to support the hypothesis derived from the objectives. The components of GLAD Back are described in Section 4 and based on outlined supporting evidence from the literature in Section 3, as well as piloting and feedback from people with back pain participating in the preliminary program and clinicians participating in the initial training and delivery of the care package (Kongsted A, Ris I, Kjaer P, Vach W, Morso L, Hartvigsen J: GLA:D® Back: Implementation of group-based patient education integrated with exercises to support self-management of back pain. Protocol for a hybrid effectiveness-implementation study, submitted and Kongsted A, Hartvigsen J, Boyle E, Ris I, Kjaer P, Thomassen L, Vach W: GLA:D® Back: Implementation of group-based patient education integrated with exercises to support self-management of back pain. Feasibility of implementation by a clinician course, submitted). More details can be found under the heading Intervention Development.

### Organisation

The planning of the GLA:D Back intervention was led by the primary working group (PK, AK, IR and JH) at the University of Southern Denmark (SDU) with the involvement of invited expert clinicians and a multidisciplinary research group of national and international experts within the field, as well as an advisory board (see Acknowledgements).

Some members of the primary working group (PK, JH) are also involved in the Horizon 2020 project selfBACK [12] that aims to develop a digital decision support system for people with back pain to facilitate, improve and reinforce self-management. One of authors involved in the expert group (AA) is leading the Swedish study implementing a similar program called the BetterBack☺ model of care [13]. The interventions of the GLA:D Back, selfBACK and BetterBack☺ are developed in parallel and share the same theoretical base and several specific components (Svendsen MJ, Sandal LF, Kjaer P, Nicholl BI, Cooper K, Holtermann A, Mair FS, Hartvigsen J, Stochkendahl MJ, Sogaard K et al: Intervention mapping for developing an app-based decision support system to improve self-management of non-specific low back pain (SELFBACK), in preparation).

### Processes

The literature reviews and drafts for Sections 1–4 (*rationale for GLA:D Back, the program objectives, the program design, theories and evidence, and the program)* were prepared by the primary working group at SDU in close collaboration with the other authors and people from the multidisciplinary expert group. This was a non-linear process involving literature reviews, group discussions, consensus processes, initial testing and pilot studies [8, 9].

### Intervention development

Section 1, *rationale for GLA:D Back,* was based on literature dealing with back pain, its consequences for the individual and the society, prognostic factors for disabling back pain as well as the challenges facing clinicians. Section 2, *program objectives* of GLA:D Back, was developed by the primary working group at SDU using an iterative process, with feedback from the expert group, and in collaboration with the selfBACK [14] and BetterBack☺ groups [13]. It included the results from the processes related to Section 3, *program design, theories and evidence*, with core elements for the intervention content extracted from clinical guidelines, reviews and randomised controlled trials and these were discussed in the multidisciplinary expert group. Inclusion criteria for the selection of components for the intervention were that they should 1) include patient education, 2) be suitable for groups of patients, 3) be targeting patients with recurrent and/or persistent non-specific back pain, and 4) address factors related to poor outcomes. Consensus on the inclusion of these components was sought over two rounds, where members of the multidisciplinary expert team gave their feedback on, and prioritised, educational aspects and exercises. The first GLA:D Back intervention was then outlined by the authors and further discussed with the multidisciplinary expert team. In Section 4, the final components of the GLA:D Back program were described as well as the testing of this program. The first version of the program was tested initially at the university clinic at SDU by PK, IR and AK and the second version in a pilot study in nine primary care chiropractic and physiotherapy clinics. The detailed results from these studies are reported in separate publications (Kongsted A, Ris I, Kjaer P, Vach W, Morso L, Hartvigsen J: GLA:D® Back: Implementation of group-based patient education integrated with exercises to support self-management of back pain. Protocol for a hybrid effectiveness-implementation study, submitted and Kongsted A, Hartvigsen J, Boyle E, Ris I, Kjaer P, Thomassen L, Vach W: GLA:D® Back: Implementation of group-based patient education integrated with exercises to support self-management of back pain. Feasibility of implementation by a clinician course, submitted).

## Results

### Rationale for GLA:D Back

#### The burden of back pain

Back pain is the most common reason for people in Denmark visiting general practitioners (GPs) [15] and it is responsible for more years lived with disability worldwide than any other condition [16, 17]. The societal, health care and economic burden associated with back pain is high and comparable to conditions such as cardiovascular disease, cancer, mental health, and autoimmune diseases [18]. In Denmark, every tenth visit to a GP and every third visit to a chiropractor or physiotherapist is due to back pain [15]. Almost one in five patients consulting a Danish GP for back pain has severe persistent pain [19]. Single episodes of back pain usually resolve quickly but recurrent episodes are very common [20–23]. Patients with persistent back pain describe the condition as negatively affecting their lives, leaving them disempowered, and that the outcomes of consultations with health care professionals are often inadequate [24]. Further, half of the patients attending a GP due to back pain believe that they need imaging [25]. *There is an obvious need to reduce the burden of back pain both in terms of the disability and poor quality of life experienced by people who live with severe back pain and in terms of the substantial costs to society.*

#### A need for care that integrates physical, psychological, and cognitive factors

The traditional biomedical approach to health care implies that a patho-anatomical or patho-physiological diagnosis needs to be established to guide the choice of treatment with the goal of curing the disease. In many non-communicable conditions, such as musculoskeletal disorders and back pain, however, this model is not very useful [16, 26]. In approximately 90% of back pain cases, a specific structural pain generator cannot be identified, and thus, a structural diagnosis cannot guide treatment decisions [16]. In addition, back pain is frequently recurrent or persistent [16] and in cases where both clinicians and patients often expect to cure the pain, expectations are not met and the diagnosis and treatment are questioned [27]. Consequently, the patient might seek another structural explanation, new testing, and a new treatment. This may explain the increased use of imaging, injections and surgery for back pain without clear targets for the interventions and without positive effects on patient outcomes [2, 28]. The biomedical approach clearly does not address the complex interplay between pain, function, self-perceived limitations, coping, societal circumstances such as labour market conditions, and an individual’s capacity to take control of their own health and cope with changing life circumstances [2, 29].

People with back pain want to be able to control their pain, receive an explanation for their pain, be given a diagnosis, benefit from pain relief, be able to manage everyday life and have a coherent concept about their pain that makes sense to them [27, 30]. These patient goals can be summarised as being able to self-manage, according to the definition we have used in this study [7].

*Models of care based only on structural biomedical beliefs are not helpful for most people with back pain, and there is a need for care based upon a biopsychosocial framework that convincingly communicates the natural course of back pain.*

#### Challenges for the clinician

Clinical guidelines consistently recommend use of patient education, a patient’s active participation, exercises, physical activity, and in some cases, manual therapy with a perspective towards self-management [1, 31, 32]*.* However, the specific content of the information, education, exercises and physical activity is so far poorly described and implemented. Clinicians dealing with patients with back pain face the challenge of addressing all the relevant components influencing persistent and recurrent back pain in the intervention, and the challenge of integrating these components may be an important factor in the evidence-practice gap [33]. Further to this, successful implementation of guideline recommendations require interventions to be accepted by patients and clinicians and to be feasible in the clinical context [34]. GPs express insecurity and lack of knowledge as a barrier to moving away from the biomedical model towards a more comprehensive bio-psycho-social model [35]. This has led to large variations in the management of patients with back pain and also to conflicting messages to patients. For example, clinician beliefs that promote physical inactivity and sick leave during episodes of back pain hinder the implementation of self-management strategies in clinical practice [36].

*There is a need for more specific descriptions of the content of patient education, exercise and self-management strategies, as well as their method of delivery, and treatment regimen to guide the provision of evidence-based advice and treatment to patients with back pain.*

#### Factors related to disabling back pain

The development of disabling back pain is a complex process affected by multiple internal and external factors of which many are interrelated, and some are modifiable [16, 37]. Socioeconomic factors and a number of comorbidities are not directly modifiable, whereas prognostic factors such as pain intensity, fear of movement, pain catastrophising, negative mood, and negative back pain beliefs may be modifiable with subsequent potential positive impact on disability [16, 38–41]. Pain catastrophising, pain control and illness perceptions are not only prognostic factors but have also been demonstrated to be potential targets of interventions that mediate the treatment effect [42–44].

Central to the development of disability is low self-efficacy (perceived inability to manage back pain) and related factors including pain distress, negative expectations about the course of back pain, fear and perceived low pain control [43–47]. This implies that viewing back pain as a purely structural problem may be unhelpful because structural injury or dysfunction is difficult to control or manage and makes people attempt to avoid stress on painful structures, and adapt their behaviours accordingly [27, 37, 48, 49]. If that adaptation involves reduced activity and less social participation, acts or behaviours to control or avoid pain may become part of the pain condition and the problem itself.

Pain behaviours are shaped by the rules of learning theory through positive and negative reinforcement [50]. For instance, negative health beliefs can lead to complaints of pain and overt expressions of pain, which may be reinforced by increased attention and assessment by health care personnel [51]. Negatively reinforced pain behaviours may develop into maladaptive coping strategies simply because they serve to decrease immediate anxiety and emotional distress.

It may be possible to support patients’ development of self-efficacy through 1) providing positive experience with performance, 2) vicarious experience by observing other people in a similar situation, 3) social persuasion, and 4) assisting patients in the interpretation of physiological feedback during activities [52].

*It is important that interventions for back pain not only focus on anatomical or physiological problems but also consider these in connection with psychological, social and behavioural aspects of the pain condition which address positive operant conditioning and the learning of adaptive self-management strategies* [53]*, while avoiding negative reinforcement of maladaptive behaviours* [54].

In persistent back pain, habitual and restricted movement patterns are often present [55]. This is an important part of disability because free movement and easy engagement in daily activities are hindered, and this may in itself maintain pain [56, 57]. Alterations to movement have been described and indicate that people with chronic back pain have less variation in movement, which can be caused by stereotypical habituated recruitment of muscle fibres or by avoidance of certain movements [56, 58]. In addition, feelings, thoughts and behaviours are closely connected, and movement patterns associated with back pain can be strongly influenced by beliefs and fears of damaging spinal structures [37, 59–63].

*Disabling back pain is related to low self-efficacy, fear of movement, negative beliefs and reduced variation in movement, all of which are interrelated. Therefore, people with persistent back pain need to be educated about these relationships to understand that back pain can be controlled and managed through new ways of thinking, movement and active living.*

#### The context for the GLA:D Back intervention

In Denmark, people with non-specific back pain are primarily managed by GPs, chiropractors, and physiotherapists, and to a smaller degree in outpatient hospital clinics and municipality rehabilitation centres. GPs, physiotherapists and chiropractors in primary care are self-employed, with GP services being fully reimbursed by the universal health insurance, and physiotherapist and chiropractic services being partly reimbursed. The target population for GLA:D Back is people with back pain consulting one of the 2850 physiotherapists or 410 chiropractors working in primary care (2017 numbers) or in the municipality rehabilitation centres. Patients are typically referred to physiotherapists from a GP or they self-refer to chiropractors. More details about the setting are provided in the GLA:D Back protocol paper (Kongsted A, Ris I, Kjaer P, Vach W, Morso L, Hartvigsen J: GLA:D® Back: Implementation of group-based patient education integrated with exercises to support self-management of back pain. Protocol for a hybrid effectiveness-implementation study, submitted).

#### Summary of rationale for GLA:D Back

The literature reviews and the discussions within the planning group led to the identification of a number of obvious needs of patients with persistent back pain, for clinicians dealing with these patients, and for society. An overview of who and what needs to change for the patients is summarised in Table 1. Primarily, there is a need for interventions that provide a change in a patient’s beliefs, feelings and behaviours associated with back pain through helpful explanations of pain that can replace purely structural explanations, help the patient with disability to restore varied movement and have confidence in movement and physical activities, all of which are intended to support a patient’s engagement with self-management strategies. Thus, GLA:D Back was developed to address prognostic factors of disabling back pain and potential treatment effect mediators by compiling elements of effective interventions into a care package that would be feasible in primary care and acceptable to patients and clinicians.

**Table 1:** An overview of identified change objectives from the literature and group discussions for people with back pain, including potential interventions and the relevant components

Who	What: Change objects	How: Interventions	Intervention components
People with back pain	Quality of life	Education, exercises and activity engagement	Sum of all components
	Self-efficacy, and sense of control	Education and exercise	Information about back pain (triggers, prognosis, treatment guidelines, imaging, structures of the back) Promotion of cognitive and behavioural strategies (balancing resources and demands, understanding pain mechanisms, exploring movements, self-management) Reassurance that pain does not mean harm Decreasing fear of movement through increased confidence in physical/back capacity
	Self-management Disability	Education and exercise	Encouragement to stay physically active and continue with normal/everyday activities Encouragement to explore different ways of moving during exercise Increasing functional activity level via progressive exercises
	Pain experience and control over pain	Education and exercise	Understanding and accepting pain Use of exercise for pain relief Identifying worsening and easing componentsPromotion of pain coping skills (e.g. goal-setting, action planning, pacing, problem solving, relaxation, distraction, graded exposure)
	Negative thoughts and beliefs	Education and exercise	Education about changing thought patterns to avoid catastrophising and negative thoughts Experiences of increased physical capacity via progressive exercisesPromotion of a positive and ‘in control’ attitude
	Structural beliefs	Education and exercise	Encouragement to stay physically active and continue with normal/everyday activities Reassurance about the favourable prognosisReassurance that pain does not mean harm
	Expectations about exact diagnosis and imaging	Education	Describing back pain as a recurrent condition Explaining that imaging is not generally recommended because findings are unspecific and do not inform care
	Better interaction with health professionals	Education	Recognition that back pain interacts with many aspects of life
	Free and natural movements	Education and exercise	Individually adapted level of exercises including strength and flexibility An approach to exercise that does not dictate one correct way Encouragement to explore variation in movement during exercise
	Fear of movement	Education and exercise	
	Muscle strength	Exercise	
	Physical fitness	Education, exercise and physical activity	Explanations of beneficial effects of physical activity and exercise

We also identified a number of change objects for the clinician and the society (see Table 2). These are further addressed in the protocol paper where the implementation is described in more detail (Kongsted 2018a, in submission).

**Table 2:** An overview of identified change objectives from the literature and group discussions for health care providers and the society including potential interventions and their relevant components

Who	What: Change objects	How: Interventions
Health care provider	Change of biomedical beliefs towards bio-psycho-social and cognitive understanding	Clinician course (see protocol paper)
	Knowledge about management of people with back pain	Clinician course (see protocol paper)
	Ways to implement a non-structural approach	Patient education materials, examples of exercise program guidance and progression
Society	Number of consultations	Sum of all components of the GLA:D Back program and the clinician course (see protocol paper)
	Amount of sick leave	
	Medication	
	Use of imaging	
	Use of surgery	
	Use of injections	

### Program objectives

#### The overall goal

The overall program goal is to reduce the burden of back pain for individuals and for society by improving patient self-management and reducing the use of health care services that are ineffective or potentially harmful [2].

#### Change objects

**Fig. 1:**
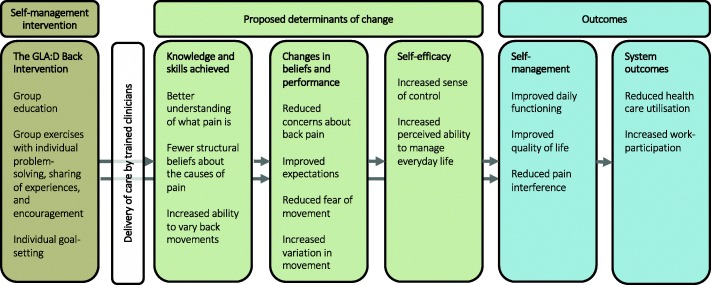
Program model. Overview of the GLA:D Back intervention, the proposed patient achievements and outcomes through the GLA:D Back program and their theoretical links

The immediate objectives of GLA:D Back are 1) to provide patients with knowledge to understand back pain as a benign and recurrent condition influenced by multiple factors, and 2) to provide patients with skills in pain management strategies, in specific back exercises and in exploring variation in movement (Fig. 1). The detailed change objects for the patients with back pain are shown in Table 1.

#### Performance and behavioural objectives

The main performance objectives are that the participating patients can self-manage their pain, have knowledge about pain and how it can be influenced and managed, have less fear of pain, have positive expectations about their back, move more freely and increase their physical and mental capacities. All performance objectives are listed in Table 3.

**Table 3:** Overview of the key messages, educational themes and performance objectives for the person with low back pain and the exercise components of the GLA:D Back intervention. The Table is constructed from the literature and consensus discussions about the content of the intervention

Key Messages	Educational theme/activity	Self-Management
		Performance objectives
A healthy back requires a balance between demands and capacity	Behavioural pain control	The person with back pain achieves an increased *sense of control* and an *ability to manage everyday* life through being able to reduce demands and increase capacity, through: ● insights into social, physical and mental factors affecting pain ● reducing monotonous loads by varied motion ● increased physical capacity
Pain = Alarm Pain ≠ Harm The brain can turn pain up and down	Pain mechanisms Exercise	The person with back pain achieves *increased understanding of pain* and *reduced fear and concerns* through: ● knowledge about pain mechanisms ● knowledge about how thoughts, fear and beliefs influence the pain experience ● knowledge that pain can be influenced by distraction, exercise and physical activity ● positive non-fearful experience with movement and exercise
Bad posture and deformations of the spine are common	Imaging Structural pain sources	The person with back pain has *fewer structural beliefs* from understanding that spinal curves, posture and imaging findings relate poorly to pain through: ● knowledge about common findings on MRI and their lack of association with back pain ● knowledge that there is a wide variation in the appearance of healthy and strong spines
Action comes before improvement Natural movements inhibit pain Exercise strengthens the back	Treatment	The person with back pain achieves *improved expectation* through: ● understanding that engaging in physical activity and work is part of the cure, rather than something to wait for until cured ● experiencing that movement reduces pain ● enhanced awareness of muscle function and perceptions of bodily control
The back is made for movement The back is strong	Exercises Physical activity	The person with back pain obtains physical skills with *variation in movement* through: ● positive experiences with movement ● exploring variation in movement ● knowledge about how exercises improve back capacity and decrease pain

We hypothesised that improved illness beliefs, increased perceived ability to perform exercises and being physically active lead to reduced fear, improved expectations and increased perceived physical capacity. These changes were then hypothesised to increase patients’ self-efficacy, which in turn was expected to improve daily functioning, quality of life and pain levels and thereby improve self-management. Finally, this was intended to translate into reduced health care utilisation and back pain-related sick leave (Fig 1). The outcome measures for the different domains are listed in Table 4.

**Table 4:** An overview of the targets for the interventions, the corresponding intervention components and outcome measures^a^

Target	Intervention component directed at the target	Measured construct (measurement tool)
Knowledge and beliefs Pain coherence Back Pain Beliefs Expectations Fear of movement	Patient education Exercise sessions Motivation to engage in general physical activity	Illness Perceptions (B-IPQ) [146, 147] Fear of movement (FABQ) [148, 149]
Skills and performance Ability to vary back movements and move freely Strength Mobility Physical capacity	Exercise sessions Motivation to engage in general physical activity	Perceived ability to perform exercises (single item) Physical fitness (Self-assessed physical capacity analogue scale) [150] Muscle endurance [135], flexibility [133, 151], and sit-to-stand [152]
Self-efficacy Perceived ability to manage pain	Patient education Exercise sessions Motivation to engage in general physical activity	Self-efficacy (Arthritis Self-Efficacy Scale) [153]
Self-management success Daily activities Quality of life Pain	Patient education Exercise sessions Motivation to engage in general physical activity	Disability (Oswestry Disability Index) [154, 155] General health, social functioning and mental health (SF-36) Pain interference (B-IPQ) [148, 149, 156] Pain intensity (NRS 0–10) [157]
System Outcomes Health care utilisation Sick leave	Patient education Exercise sessions Motivation to engage in general physical activity	Visits to health care providers, imaging, pain medication and sick leave (Danish national registries) [158, 159]

^a^Details of the effect, evaluation and outcome measures are reported elsewhere (Kongsted A, Ris I, Kjaer P, Vach W, Morso L, Hartvigsen J: GLA:D® Back: Implementation of group-based patient education integrated with exercises to support self-management of back pain. Protocol for a hybrid effectiveness-implementation study, submitted)

### Program design: Hypothesis, theories and evidence

In this section, we present hypotheses, theories and evidence to support the GLA:D Back intervention focusing on the following six topics: self-management, the cognitive approach, education for back pain, exercises for back pain, combined education plus exercise for back pain, and pedagogic considerations.

#### Self-management

We hypothesised *that increased knowledge and improved cognitive and physical skills would help reduce the negative impact of back pain through an increased ability to manage everyday life reflected in improved self-efficacy, less disability and less need for health care (*Table 3*).*

The overall focus of GLA:D Back is to increase self-management, which is pursued through activities designed to increase self-efficacy as shown in Table 5 [52].

**Table 5:** Key activities for supporting self-efficacy according to social cognitive theory [52]

Determinants of self-efficacy	Presence in GLA:D Back	Activity in GLA:D
Performance accomplishments	Positive experiences with movement	Focus point of exercise delivery
Vicarious experience	Observing and interacting with fellow patients	Group-based intervention
Social persuasion	Verbal encouragement during exercises	Motivating patients to explore movement rather than correcting performance
Physiological feedback	Providing non-threatening explanations for pain provocation during movement	Integrating education on pain mechanisms with exercise supervision (Additional file 1)

Core elements of self-management interventions are patients engaging in their own care and health-promoting activities, setting goals with the clinician who can help them to make informed choices, and being empowered through knowledge about the condition [64–66]. This has previously been pursued by combining elements of patient education, physical activity, exercises and cognitive therapy [67]. Overall, such programs have shown small to moderate effects on pain, disability and self-efficacy, whereas there is no consensus on direct measures of the ability to self-manage [64, 65]. A clear limitation of most interventions that were described as self-management interventions for back pain is the lack of a theoretical base [65], and importantly, no evident attempt to integrate educational elements and physical activities or exercises [64]. In contrast, this integration has been a focus of Cognitive Functional Therapy (CFT) [37].

#### The cognitive approach

We hypothesised that *changes in beliefs and performances would increase patients’ ability to self-manage their back problem.*

The model to facilitate self-management and behavioural change is based upon the Cognitive Behaviour Therapy (CBT) model [68]. The model outlines how cognitive appraisals, i.e. pain catastrophising and poor health beliefs negatively influence feelings, bodily sensations and behaviours, a process that is reciprocal and often leads to individuals being caught in vicious cycles and maladaptive pain behaviours. This vicious cycle is well described in the very influential ‘fear-avoidance’ model of chronic back pain [59]. Also, pain relief or pain control may for some patients become such a dominating or salient goal that it interferes with other valuable life goals resulting in activity avoidance [62]. In this case, psychoeducation in the model of pain being a dynamic system affected by cognitions, emotions and behaviours, combined with group exercises in problem-solving skills and graded exposure to important everyday life activities, is a means to restore activity engagement. Hence, patient education with a CBT approach should evolve around psychoeducation about pain, and the promotion of pain coping skills like activity pacing and progression guidance, goal-setting, action planning and relaxation techniques [69]. Following this concept, GLA:D Back intends to reinforce healthy behaviours and reduce pain behaviours by using key messages in pain education focused on creating positive expectations such as *hurt does not equal harm* and *movement inhibits pain* in addition to goal-setting.

There is evidence from several reviews that CBT approaches addressing risk factors such as fear-avoidance beliefs and pain-catastrophising can improve fear-avoidance beliefs, pain, disability and quality of life in comparison with no treatment or usual care [70–77]. One review showed high-quality evidence that CBT interventions provided by trained physiotherapists and delivered within a physiotherapy setting were more effective than other guideline-based treatments [70]. However, there is inadequate information in the studies on how the evidence-based CBT interventions were implemented in clinical practice. Operant conditioning has been shown in a systematic review to be a promising CBT-based strategy for the prevention of chronic back pain [54].

#### Education for back pain

The performance objectives related to education are listed in Table 3 and we proposed two different hypotheses with their underlying theories and evidence:

*We hypothesised that : a) a person with back pain would be able to manage his/her pain through knowledge about non-specific back pain, its pain mechanisms, sources, trajectories and prognosis and thereby change back pain beliefs, expectations and fear of movement, and b) a person with back pain would be able to understand and control his/her pain through knowledge about pain mechanisms, knowledge about how thoughts, fear and beliefs influence the pain experience, knowledge about pain being influenced by distraction, exercise and physical activity, positive non-fearful experience with movement and exercise, staying active and adapting activities, thereby changing back pain beliefs, and fear of movement.*

The most consistent themes in education in the clinical guidelines involve information about what back pain is and what can be expected in the future; reassurance; understanding and accepting pain; avoiding catastrophising and negative thoughts; and encouragement to stay physically active and continue with normal activities including work [1, 31, 32, 69, 78–80]. However, the completeness in the reporting of the content is poor and often not in accordance with the underlying randomised controlled trials [81]. From pain science, it is well-established that cognitions and feelings affect central pain modulation and thereby pain intensity [82] and the relationship is bidirectional with the pain experience also affecting thoughts, beliefs and feelings, such as fear avoidance beliefs, hypervigilance, expectations, and anxiety [41, 83].

Response to pain is influenced by beliefs about it and its emotional significance [79], shaped by our memories and prior experience [50, 84]. Most importantly, beliefs are about the nature of pain, self-efficacy, and the consequences of harm and further injury [85]. Also, attitudes and beliefs of the health care practitioners influence patients’ beliefs and are significantly associated with the health care practitioners’ advice and recommendations and treatment decisions [84].

Despite patient education being promoted in all guidelines [1], the evidence for its effect is sparse and conflicting [86]. One systematic review that included only two studies with very low-quality evidence, suggested education in pain neurophysiology to be a promising intervention for the primary outcome measures of pain, physical, psychological and social function [71], while a more recent review has shown there is moderate to high-quality evidence that patient education in primary care can provide long-term reassurance for patients with acute or subacute low back pain [87].

#### Exercise for back pain

We hypothesised that *people with back pain would improve the variability of their individual back movements and move more freely through knowledge about the effects of different types of exercises, goal-setting, exercises for strength, flexibility, exploring movement, and motivation for activities to improve physical capacity* (Table 2).

The exercise program is based on theories about changes in physical functioning [88], neuromuscular changes [89], decrease in physical fitness [90] as well as altered patterns of activity [91] and levels of activity [92] as reported in people with back pain. Uniform and restricted movement patterns are often present in people with back pain [55–57]. Alterations of movement have been measured and described in various ways and indicate that people with chronic back pain have reduced variation in movement, which can be within the muscle as stereotypical habituated recruitment of muscle fibres or it can be by avoidance of certain movements [56, 58]. This may lead to deconditioning [93], where people with back pain are restricted in performing everyday physical activities and at higher risk of developing an inactive lifestyle. This may set up a vicious cycle of inactivity where a reduction in physical fitness (deconditioning) leads to further reduced activity.

Exercises should be tailored to the individual and consider the individual’s thoughts, beliefs, fears, motivation and previous experience, as well as physical capacity and confidence in exercising.

Generally, exercise therapy is recommended for reducing musculoskeletal pain [94], and there is substantial evidence from systematic reviews to support exercise therapy as an effective [95–101] and cost-effective [102] intervention for reducing pain and improving function and quality of life in people with back pain. Four systematic reviews have addressed therapeutic exercise as a prevention strategy, either as a post-treatment intervention or as a particular part of the intervention focusing on prevention of recurrence and duration of new episodes [95, 103–105]. Positive effects have been reported for different types of exercise therapy spanning general strengthening [101, 106–108], endurance training [109], direction-specific repeated movements and flexibility [99, 100, 110, 111], yoga, Pilates [108, 112], and motor control exercises [96, 97, 99, 113, 114] with a focus on specific muscles such as the transverse abdominal or multifidus muscles as compared with no treatment or ‘usual care’ [115]. No single form of exercise is clearly superior to any other, but exercise seems to be more effective in people with chronic and persistent back pain than in people with back pain of shorter duration [98].

The dose of exercise in primary randomised controlled trials included in the reviews is not always clearly described. In an early review of exercise for back pain, an average of 16 weeks of exercise was identified from 61 studies, but weekly frequency was not reported [111]. Although inconclusive, it appears that longer durations of exercise periods and heavier training is more effective in reducing back pain when compared with shorter periods and lighter loads [106]. Based on an average duration of 8 weeks for interventions reported in a systematic review of programs aiming to develop patient self-management for chronic low back pain [64], as well as consensus within the multidisciplinary expert team and feedback during the piloting of the care package, an 8-week intervention period with a total of 16 sessions was considered adequate to support the aims of the care package and the needs of patients and care givers. The American College of Sports Medicine recommends 2–3 weekly sessions for muscle training at 60–70% of one repetition maximum (RM) for novice trainers and 80% of 1RM for experienced people, sets of 8–12 repetitions for strength and power and > 15 for endurance [116]. To maintain good range of motion, flexibility exercises to end range are recommended 2–3 days a week, held for 30 s and repeated 2–4 times [116]. The recommendations for improving cardiovascular fitness are 5 days/week of moderate exercise (Borg ratings of perceived exertion (RPE) 12–16) 30-60 min/day, or 3 days/week of vigorous exercise (Borg RPE 17–20) 150 mins/week, or a combination of moderate and vigorous exercise 3–5 days/week [116].

#### Combined interventions

We hypothesised *that a close integration of the key messages from patient education including knowledge about pain mechanisms and addressing pain-related fears in the exercise sessions would improve the outcomes for patients more than stand-alone interventions*. This is supported by the previously outlined theories and emerging evidence of positive outcomes on function and pain in the literature [37, 117–123].

By also implementing the theory of operant conditioning in the exercise sessions, GLA:D Back intends to reinforce healthy behaviours and reduce pain behaviours by using an exercise quota for increasing general activity levels, which is gradually built up towards a realistic predefined goal [124]. The key messages from pain education will be repeated during the exercise sessions to increase self-management (Table 2).

More recent papers, for example on CFT, emphasise the potential of combining educational, cognitive and exercise approaches [37, 117–121] and a randomised controlled trial has shown larger effect sizes for pain reduction and functional limitations than are normally seen in interventions for back pain [122, 123]. New models of care, where patients are stratified to a combined exercise and cognitive approach for the most severe incidences of back pain, have shown promising results and, in particular, to be cost-saving because of the reduction in unnecessary treatments [122]. Another recent example of effective and combined interventions is the Back Skills Training Trial where a structured group-based program based on a multidisciplinary cognitive behavioural intervention showed better outcomes in functional limitation from this intervention arm [125] as well as improved cost-effectiveness [126].

A recent review also showed the largest reduction (45%) in new episodes of back pain with interventions combining exercise and patient education [95].

The duration of combined interventions varies between 6 and 12 weeks [122, 123, 125].

#### Pedagogic consideration

We hypothesised that *an active learning environment with patient involvement would be needed to achieve reflection on thoughts, beliefs and behaviours.*

For patients to change thoughts, beliefs and behaviours, we considered several theories about achieving self-efficacy and behavioural change [10, 52], individual learning strategies [127, 128], problem-based learning [129], and how to deliver the interventions. The program was built on the basic idea of creating a learning environment where there is capacity for patients to try to formulate and discuss both the theory and practice of doing exercise in an interactive and supportive context [130]. Despite the poor scientific support for different learning styles [127, 128], these theories were taken into account by using both audio and visual presentations during patient education, and taking the key messages to the practical sessions, where they were repeated while doing exercises in order to create an embodied experience [131, 132].

### The program

The program presented here is the final version of GLA:D Back after implementing feedback from the initial testing and pilot studies.

#### Program description

GLA:D Back includes an initial individual testing session, two group sessions of patient education, 16 bi-weekly one-hour sessions of supervised group exercises, and a final individual testing session. This structure is identical to the original GLA:D knee and hip program, with the exception of the length of the exercise program being 8 weeks compared with 6 weeks for GLA:D knee and hip [4].

#### Individual testing sessions and goal-setting

At the individual testing session, the patient is registered in the database and results from the performance tests: standing forward bending test [133], back extensor endurance test [134, 135], trunk flexor endurance test [135, 136] and the sit-to-stand test [137, 138] are recorded electronically. Instructions including detailed descriptions and photos are made available to the GLA:D Back clinician.

The database is a key element in GLA:D Back and it is a requirement for all participating clinicians and patients to enter their data. After the clinician registers the patients, they receive a link to the database by email. This opens the baseline registration of key information about the patients and the automatic follow up at 3, 6 and 12 months on the patients’ outcomes. For more details about the database and outcome measures (Kongsted A, Ris I, Kjaer P, Vach W, Morso L, Hartvigsen J: GLA:D® Back: Implementation of group-based patient education integrated with exercises to support self-management of back pain. Protocol for a hybrid effectiveness-implementation study, submitted). 

The level at which to start the exercises is explored by the patient in collaboration with the clinician. For each of the eight types of exercise (see below), the difficulty for the patient is discussed in order to give the patient an idea of the entry level for each exercise type in the group sessions.

During the initial session, the patient and the clinician discuss the patient’s goals with respect to participating in the program. The goal-setting session is inspired by the Specific, Measurable, Attainable, Realistic Timed (SMART) concept [124]. Goals related to function and participation in everyday life are formulated, written on the patient’s personal exercise program, and entered into the database by the clinician. Furthermore, the number of weeks, time or repetitions to reach the goal and the acceptable level of discomfort related to attaining the goal are recorded.

At the final individual testing session, after completing the program, the performance tests are repeated, and the goal attainment is evaluated. The results are entered into the database by the clinician.

#### Patient education

**Fig. 2:**
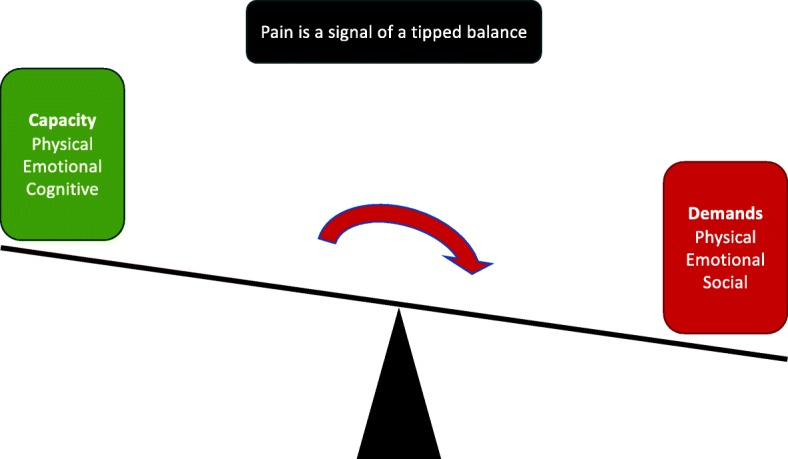
The balance model. Illustration from the patient education explaining that pain is a result of your demands (physical, emotional and social) exceeding your capacity (physical, emotional, and cognitive)

**Fig. 3:**
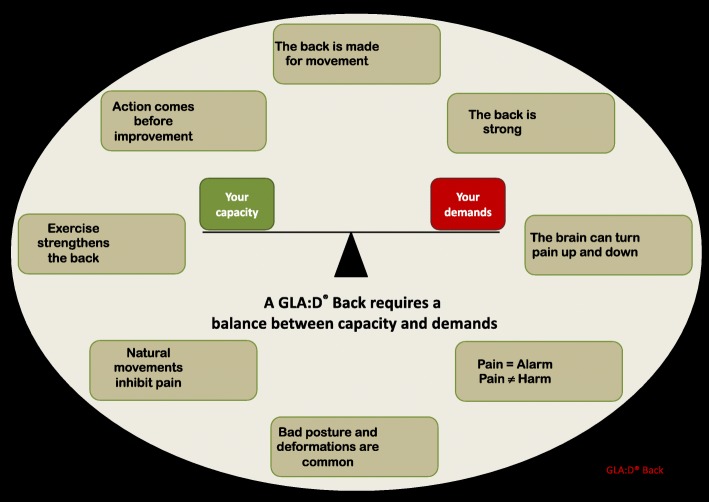
Key messages in GLA:D® Back. An overview of key messages from the GLA:D Back pain education material. (GLA:D^®^ is a registered Trademark of the University of Southern Denmark: The name can only be used for an intervention if all criteria described by the University of Southern Denmark are met).

The two educational sessions address the balance between demands and capacity (Fig. 2), the causes and clinical course of back pain, symptoms, need for imaging, treatment options, pain explanations, management of pain, and first aid for back pain. The content has been extracted from patient education used in randomised controlled trials (RCT) and builds on the previously identified needs of the patients, modifiable risk factors, as well as the proposed change objects (Table 1) and performance objectives (Table 3 and Fig. 1). For this, the primary working group produced two PowerPoint (PPT) slide presentations with full manuscripts to be used by treating clinicians. As a supplement to the PPT slides, paper-based information summaries were produced for patient involvement and engagement in the educational sessions, for example, for the patient to consider factors that increase or reduce the pain experience. The key messages (Table 3 and Fig. 3) were printed on a poster to be used during the education and exercise sessions. The PPT slides and related manuscript are made available to GLA:D Back providers online and examples are printed for the clinician course.

#### Exercise sessions

**Fig. 4:**
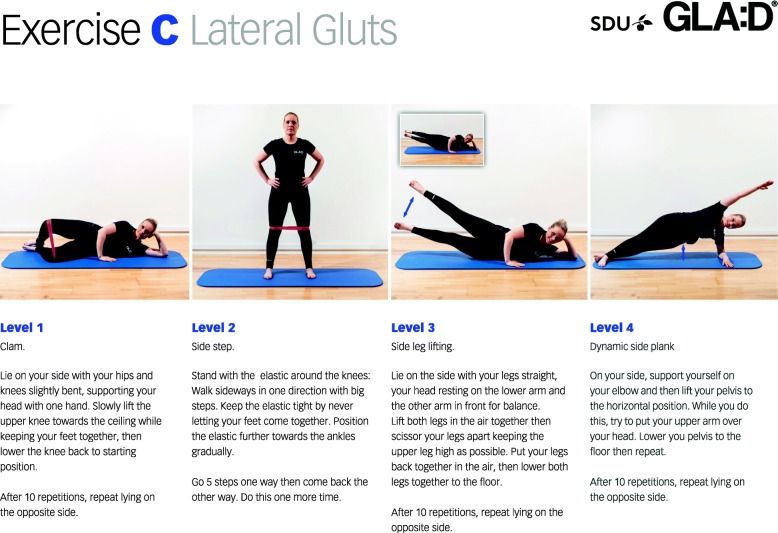
Exercise example. An example of exercises for the lateral buttocks at four different levels. The individual depicted in the images provided her written informed consent for the publication of this identifiable image. (GLA:D^®^ is a registered Trademark of the University of Southern Denmark: The name can only be used for an intervention if all criteria described by the University of Southern Denmark are met).

The exercise program includes a warm-up session of five exercises (awareness of the back, pelvic tilt, lumbar rotation, arm movements, whole-body movement in standing), well known exercises targeting the muscles of the back extensors, abdominals, lateral buttocks, trunk rotators, posterior buttocks, leg muscles, oblique abdominals (e.g. the plank, diagonal arm and leg lift), as well as exercises for flexibility (Additional file 1). For each of the eight types of exercise, four different levels of difficulty are shown, including photos and written instructions (see example shown in Fig. 4 and an overview of all exercises in Additional file 1). After each type of exercise, a diary is available where the patient records the level of each exercise for each training session during the eight-week period. The program ends with examples of eight different stretching exercises shown as photographs and written instructions.

The written exercise program is used throughout the 16 sessions of exercise over 8 weeks and the patients are encouraged to use it at home. The average duration of interventions reported in a systematic review of programs aimed at developing patient self-management for chronic low back pain support this duration of intervention [64]. As a supplement to the exercise program, we produced posters showing all exercises to be placed in the training facilities for an easy overview (Additional file 1).

Each exercise session includes discussions with the patients about experiences since the previous session, goal attainment, physical activity, elements from the patient education, a short warm-up session, two to three sets of the eight types of exercises, aiming for between 8 and 12 repetitions of each, and an optional short stretching session at the end of the session. The stretching part was optional due to time constraints and because stretching exercises on their own were not considered important. The patient works at his/her own level at each type of exercise and is encouraged to take responsibility for progression by evaluating his/her own performance and recording the level of the exercise, the number of repetitions in each set and the number of sets for each exercise. When a target of three sets of 10 repetitions is reached, the patient is encouraged to continue to the next and more difficult level and if this is too hard, to stay at the same level, and on bad days, to perform the exercise at a lower level. The clinician guides the patient to ensure that movements judged to be impaired or avoided are not resumed and habitual uniform inappropriate movement patterns are challenged.

During exercise sessions positive and negative pain responses explored by the participants are addressed with curiosity and seen as a potential means for learning how to manage pain, by doing more, doing less, doing the exercise differently, doing deep breathing or doing another exercise and exploring new ways to move that are less painful. Also, emphases are placed on the fact that none of the exercises can damage or harm the back. One review explored whether exercises for musculoskeletal pain should be painful or not [94]. However, only one study concerned back pain and there were no differences in pain outcomes for those who trained with pain and those who did not [139].

#### Integrating educational components in exercise sessions

To facilitate the learning experience, the clinician directs the patient’s attention towards the bodily experience when doing the exercises, exploring variation in performing the movements rather than doing the exercises in a standardised and ‘correct’ manner, and to move the patient’s focus away from paying attention to the pain. To support the delivery of exercises within this context, a document with the central messages for the training sessions was developed that includes the primary foci during the exercises: *confidence* in performing the exercises, *enjoying movement*, *management of pain provocation*, *reasons for pain*, and *ownership of exercises*. This approach is aimed at reducing the patient’s dependency on clinicians and to facilitate the patient’s feeling of competence in managing his/her own exercises now and into the future (For more details, see Additional file 2).

To facilitate the proposed learning objectives from the patient education sessions, we extracted eight different themes from the patient education material: *posture and spinal abnormalities*; *pain equals alarm* - *not harm*; *the spine is made for movement*; *natural movements inhibit pain*; *training strengthens the back*; *action precedes improvement*; *the back is strong*; and *the brain can turn the pain up or down* (Fig. 2). These are introduced with reference to specific slides of the patient education PPT presentations and suggestions for introducing the theme and for practical implementation in the training session. The themes were printed on single sheets with a key message on one side and facilitating questions on the other side, as well as an A0 poster including all messages and the balance model (Fig. 2).

#### Initial testing

In the initial testing, the first version of GLA:D Back was delivered to a group of eight people with persistent back pain at SDU. In order to collect information for the program development, patients were interviewed about their back pain and filled in questionnaires before and after the intervention. The education sessions were recorded on video and a person from the research group was present to observe the two lectures. This, together with patient feedback, informed adjustments to the content and the pedagogic methods used. During the 16 exercise sessions, instructors experimented with delivery methods and patient responses were recorded each time to inform further adjustments to the content and the delivery of the exercises.

#### Pilot study

A pilot study was conducted at five physiotherapy and four chiropractic clinics geographically spread over Denmark (Kongsted A, Hartvigsen J, Boyle E, Ris I, Kjaer P, Thomassen L, Vach W: GLA:D® Back: Implementation of group-based patient education integrated with exercises to support self-management of back pain. Feasibility of implementation by a clinician course, submitted). At these locations, the clinical registry, the patient education program, and exercise programs were tested under real-life circumstances. A focus group interview with five clinicians and a feedback meeting was conducted with input from eleven clinicians. Furthermore, the outcomes of patients participating in the GLA:D Back program were compared with those of a group of patients with persistent back pain seen in the same clinics before the implementation. The detailed results for patients and clinicians are reported in a separate publication (Kongsted A, Hartvigsen J, Boyle E, Ris I, Kjaer P, Thomassen L, Vach W: GLA:D® Back: Implementation of group-based patient education integrated with exercises to support self-management of back pain. Feasibility of implementation by a clinician course, submitted). The pilot study resulted in adjustments to the content and layout of the course material, but no major changes in the overall program.

#### Adjustments to the program

The content of GLA:D Back is expected to undergo continuous refinements during and beyond the implementation period. The core elements that are not subject to change are the structure of the program with individual sessions in the beginning and at the end of the program, patient education and supervised exercises, the key message that pain is not a sign of harm, the use of a behavioural model to explain the balance between demands and capacity rather than emphasising tissue damage to explain pain, and exercises delivered in such a way as to explore movement rather than to perform them in a standardised manner. The clinician and the patients are obliged to register data and outcomes in the clinical registry.

## Discussion

This paper describes how we developed the GLA:D Back program for people with persistent or recurrent back pain including its underlying theories and scientific evidence. The overarching aim of the program is to improve the ability of people with persistent or recurrent back pain to self-manage. The elements of GLA:D Back target factors that broadly affect prognosis for pain, activity limitation and deconditioning, and these elements are well suited to self-management. Thus, the novelty as compared with existing self-management interventions for back pain is the integration of patient education and exercise therapy that includes a clear aim to address known prognostic factors for developing back pain related disability. In addition to this, it was a strong focus to make the intervention feasible and acceptable for delivery in primary care after a short training course, and furthermore, that registration of patient outcomes in a clinical registry is a mandatory part of the program. Importantly, contrary to most existing programs, links to theories and existing evidence are made explicit during the course, in education material and in publications such as this one.

Reasons for creating the GLA:D Back included requests from clinicians due to the success of the GLA:D knee and hip program [5], and our intention to develop an evidence-based care package based on the most recent clinical guidelines available to patients and clinicians. We reviewed and analysed the scientific literature about back pain, its clinical course, related disability, prognostic factors, and qualitative studies about the challenges faced by clinicians when managing people with back pain. We involved clinicians in the reference group but did not systematically study clinicians’ need prior to developing the program. Also, within the multidisciplinary research expert group, many different professions were represented both as clinicians and researchers.

The burden of back pain disability is evident worldwide, not just in Denmark [15–18]. Reducing this burden will not be achieved by GLA:D Back alone. There is an urgent need for system changes and an even larger-scale implementation of evidence across professions and sectors. We could have involved in the design of GLA:D Back more diverse health system stakeholders from the Danish regional health authorities, politicians and professional health care organisations, who are responsible for organising and delivering health care in Denmark. However, our experience has been that this can often be challenging when discussions regress to being about managerial, budget and professional political interests. During the process, we were approached by one regional health authority and we arranged meetings that included representatives from GPs, chiropracto rs and physiotherapists from the five regions of Denmark. This has resulted in a continuing and positive dialogue with the regions and the health care provider representatives. We believe that this on-going dialogue has eventuated because we intended to develop and offer courses in GLA:D Back regardless of objections from administrators or professional organisations with vested political interests.

GLA:D Back is unique as a group intervention because of its close integration of patient education and exercise using an individualised cognitive approach, which is driven by the patient’s personal goals and capacities. In the literature, combined and individualised multifaceted interventions seem to have superior outcomes when compared with interventions that have single-facetted interventions [37, 95, 117–123, 125, 126]. Therefore, we designed the program to implement the key messages from the educational sessions into the exercise sessions. This was possible because GLA:D Back is founded on social cognitive theory, cognitive behavioural theory [68], operant conditioning [53], and behavioural change theories [69], where patients face their individual challenges using an exploratory approach and actively participate in tasks during both the education and exercise sessions.

These elements could have been introduced in different ways. We chose to adapt the framework of the GLA:D program for knee and hip pain because this framework has been successful and is well known to clinicians in Denmark [5]. The GLA:D framework includes three mandatory elements: 1) a course for clinicians, 2) education and supervised exercise for patients and 3) evaluation using data gathered via a registry.

The GLA:D Back program is a generic care package potentially implementable in different health systems. Similar principles have been applied in the Swedish BetterBack☺ model of care [13] and the Horizon 2020 project selfBACK (Svendsen MJ, Sandal LF, Kjaer P, Nicholl BI, Cooper K, Holtermann A, Mair FS, Hartvigsen J, Stochkendahl MJ, Sogaard K et al: Intervention mapping for developing an app-based decision support system to improve self-management of non-specific low back pain (SELFBACK), in preparation) [12], which provide potential for comparing future research outcomes. However, the content of the BetterBack☺ model of care is specifically adapted to the Swedish health care system while the selfBACK intervention is delivered using smartphone technology.

Other studies have already developed self-management programs that target psychosocial factors in chronic low back pain, for example, `Back on Track´ [140], and for osteoarthritis and low back pain in the `SOLAS´ study [141]. Both studies have outlined comprehensive theory for their intervention components with particular focus on education that addresses modifiable risk factors. Clinical trials are planned in both studies [142, 143] but so far, we have only seen promising results from the feasibility of the clinician training [144]. These studies inform and support our development of the GLA:D Back intervention. However, we believe that stronger integration between the theoretical components of patient education and performing higher dose individualised exercises will improve the probability of success with our intervention.

There is no generally agreed instrument designed to measure self-management. However, a very recent review identified 14 different proxy measures in 25 RCTs for self-management of which self-efficacy was the most common [145], although self-efficacy and self-management are different constructs.

GLA:D Back is built on the best available and generally recommended evidence for the management of people with persistent or recurrent back pain [1]. All the components of education, exercise and cognitive approaches included in GLA:D Back have been evaluated and found effective in numerous clinical trials. GLA:D Back has not been tested for effectiveness in a randomised clinical trial prior to implementation in Denmark but we have set up an ambitious implementation and evaluation plan to document the effects for individuals and society (Kongsted A, Ris I, Kjaer P, Vach W, Morso L, Hartvigsen J: GLA:D® Back: Implementation of group-based patient education integrated with exercises to support self-management of back pain. Protocol for a hybrid effectiveness-implementation study, submitted). Furthermore, plans for randomised controlled trials are underway in Canada and Australia and these will, together with the implementation and outcomes research, inform future revisions and modifications of the program.

## Conclusion

The GLA:D Back program for people with persistent or recurrent non-specific back pain includes two lectures of patient education and 16 twice-weekly exercise sessions. The content is aligned with clinical guideline recommendations and elements compiled from the underlying scientific literature about patient education, exercises and prognostic factors. It is an evidence-based program based on Social Cognitive Theory targeting patients’ goals, while considering their individual capacity for performance. The program will be implemented in Denmark in 2018 and the effects will be monitored at the individual and societal levels using data gathered via a clinical registry.

## Additional files

Additional file 1: GLA:D® Back Exercise program. The individual depicted in the images provided her written informed consent for the publication of these identifiable images (GLA:D® is a registered Trademark of the University of Southern Denmark: The name can only be used for an intervention if all criteria described by the University of Southern Denmark are met). (PDF 650 kb)

Additional file 2: Suggestions for different types of instruction for the exercises. (DOCX 42 kb)

## Abbreviations

B-IPQ: Brief Illness perceptions questionnaire
GLA:D^®^: Good Life with osteoArthritis in Denmark
GP: General practitioner
PPT: PowerPoint presentations
RCT: Randomised controlled trail
RPE: Ratings of perceived exertion

## References

[1] Oliveira CB, Maher CG, Pinto RZ, Traeger AC, Lin CC, Chenot JF, van Tulder M, Koes BW. Clinical practice guidelines for the management of non-specific low back pain in primary care: an updated overview. Eur Spine J. 2018;[Epub ahead of print]. 10.1007/s00586-018-5673-2 29971708

[2] Foster NE, Anema JR, Cherkin D, Chou R, Cohen SP, Gross DP, Ferreira PH, Fritz JM, Koes BW, Peul W (2018). Prevention and treatment of low back pain: evidence, challenges, and promising directions. Lancet.

[3] Buchbinder R, van Tulder M, Oberg B, Costa LM, Woolf A, Schoene M, Croft P (2018). Lancet low Back pain series working G: low back pain: a call for action. Lancet.

[4] Roos EM, Barton CJ, Davis AM, McGlasson R, Kemp JL, Crossley KM, Liu Q, Lin J, Skou ST. GLA:D to have a high-value option for patients with knee and hip arthritis across four continents: good life with osteoArthritis from Denmark. Br J Sports Med. 2018;[Epub ahead of print]. 10.1136/bjsports-2017-098904 29514823

[5] Skou ST, Roos EM (2017). Good life with osteoArthritis in Denmark (GLA:D): evidence-based education and supervised neuromuscular exercise delivered by certified physiotherapists nationwide. BMC Musculoskelet Disord.

[6] Roos EM, Skou ST, Grønne DT: GLA:D®. Annual Report 2017. In*.* Glaid.dk; 2017.

[7] Carnes D, Homer KE, Miles CL, Pincus T, Underwood M, Rahman A, Taylor SJ (2012). Effective delivery styles and content for self-management interventions for chronic musculoskeletal pain: a systematic literature review. Clin J Pain.

[8] Bartholomew LK, Parcel GS, Kok G (1998). Intervention mapping: a process for developing theory- and evidence-based health education programs. Health Educ Behav.

[9] Kok G, Gottlieb NH, Peters GJ, Mullen PD, Parcel GS, Ruiter RA, Fernandez ME, Markham C, Bartholomew LK (2016). A taxonomy of behaviour change methods: an intervention mapping approach. Health Psychol Rev.

[10] Michie S, van Stralen MM, West R (2011). The behaviour change wheel: a new method for characterising and designing behaviour change interventions. Implement Sci.

[11] Silva MN, Marques MM, Teixeira PJ. Testing theory in practice: The example of self-determination theory-based interventions. Eur Health Psychologist. 2014;16(5):171–180.

[12] Mork PJ, Bach K (2017). SelfBACK, a decision support system for self-management of low back pain, HORIZON2020. Impact.

[13] Abbott A, Schroder K, Enthoven P, Nilsen P, Oberg B (2018). Effectiveness of implementing a best practice primary healthcare model for low back pain (BetterBack) compared with current routine care in the Swedish context: an internal pilot study informed protocol for an effectiveness-implementation hybrid type 2 trial. BMJ Open.

[14] Mork PJ, Bach K (2018). A decision support system to enhance self-Management of low Back Pain: protocol for the selfBACK project. JMIR Res Protoc.

[15] Flachs EM, Eriksen L, Koch MB, Ryd JT, Dibba E, Skov-Ettrup L, Juel K (2015). Sygdomsbyrden i Danmark – sygdomme.

[16] Hartvigsen J, Hancock MJ, Kongsted A, Louw Q, Ferreira ML, Genevay S, Hoy D, Karppinen J, Pransky G, Sieper J (2018). What low back pain is and why we need to pay attention. Lancet.

[17] DALYs GBD, Collaborators H (2016). Global, regional, and national disability-adjusted life-years (DALYs) for 315 diseases and injuries and healthy life expectancy (HALE), 1990-2015: a systematic analysis for the global burden of disease study 2015. Lancet.

[18] Maniadakis N, Gray A (2000). The economic burden of back pain in the UK. Pain.

[19] Kongsted A, Kent P, Hestbaek L, Vach W (2015). Patients with low back pain had distinct clinical course patterns that were typically neither complete recovery nor constant pain. A latent class analysis of longitudinal data. Spine J.

[20] Lemeunier N, Leboeuf-Yde C, Gagey O (2012). The natural course of low back pain: a systematic critical literature review. Chiropract Man Thera.

[21] da Silva T, Mills K, Brown BT, Herbert RD, Maher CG, Hancock MJ (2017). Risk of recurrence of low Back pain: a systematic review. J Orthop Sports Phys Ther.

[22] Kongsted A, Hestbaek L, Kent P (2017). How can latent trajectories of back pain be translated into defined subgroups?. BMC Musculoskelet Disord.

[23] Kongsted A, Kent P, Axen I, Downie AS, Dunn KM (2016). What have we learned from ten years of trajectory research in low back pain?. BMC Musculoskelet Disord.

[24] MacNeela P, Doyle C, O'Gorman D, Ruane N, McGuire BE (2015). Experiences of chronic low back pain: a meta-ethnography of qualitative research. Health Psychol Rev.

[25] Latthe P, Latthe M, Say L, Gulmezoglu M, Khan KS (2006). WHO systematic review of prevalence of chronic pelvic pain: a neglected reproductive health morbidity. BMC Public Health.

[26] Croft P, Altman DG, Deeks JJ, Dunn KM, Hay AD, Hemingway H, LeResche L, Peat G, Perel P, Petersen SE (2015). The science of clinical practice: disease diagnosis or patient prognosis? Evidence about "what is likely to happen" should shape clinical practice. BMC Med.

[27] Bunzli S, Smith A, Schutze R, O'Sullivan P (2015). Beliefs underlying pain-related fear and how they evolve: a qualitative investigation in people with chronic back pain and high pain-related fear. BMJ Open.

[28] Deyo RA, Mirza SK, Turner JA, Martin BI (2009). Overtreating chronic back pain: time to back off?. J Am Board of Family Medicine : JABFM.

[29] Huber M, Knottnerus JA, Green L, van der Horst H, Jadad AR, Kromhout D, Leonard B, Lorig K, Loureiro MI, van der Meer JW (2011). How should we define health?. BMJ.

[30] Bunzli S, Smith A, Schutze R, Lin I, O'Sullivan P (2017). Making sense of low Back pain and pain-related fear. J Orthop Sports Phys Ther.

[31] Low back pain and sciatica in over 16s: assessment and management [https://www.nice.org.uk/guidance/NG59/chapter/Recommendations].

[32] Stochkendahl MJ, Kjaer P, Hartvigsen J, Kongsted A, Aaboe J, Andersen M, Andersen MO, Fournier G, Hojgaard B, Jensen MB (2018). National Clinical Guidelines for non-surgical treatment of patients with recent onset low back pain or lumbar radiculopathy. Eur Spine J.

[33] Synnott A, O'Keeffe M, Bunzli S, Dankaerts W, O'Sullivan P, O'Sullivan K (2015). Physiotherapists may stigmatise or feel unprepared to treat people with low back pain and psychosocial factors that influence recovery: a systematic review. J Physiother.

[34] Nilsen P (2015). Making sense of implementation theories, models and frameworks. Implement Sci.

[35] Breen A, Austin H, Campion-Smith C, Carr E, Mann E (2007). “you feel so hopeless”: a qualitative study of GP management of acute back pain. Eur J Pain.

[36] Buchbinder R, Staples M, Jolley D (2009). Doctors with a special interest in back pain have poorer knowledge about how to treat back pain. Spine (Phila Pa 1976).

[37] O'Sullivan PB, Caneiro JP, O'Keeffe M, Smith A, Dankaerts W, Fersum K, O'Sullivan K (2018). Cognitive functional therapy: an integrated behavioral approach for the targeted Management of Disabling low Back Pain. Phys Ther.

[38] Wertli MM, Rasmussen-Barr E, Held U, Weiser S, Bachmann LM, Brunner F (2014). Fear-avoidance beliefs-a moderator of treatment efficacy in patients with low back pain: a systematic review. Spine J.

[39] Wertli MM, Rasmussen-Barr E, Weiser S, Bachmann LM, Brunner F (2014). The role of fear avoidance beliefs as a prognostic factor for outcome in patients with nonspecific low back pain: a systematic review. Spine J.

[40] Campbell P, Bishop A, Dunn KM, Main CJ, Thomas E, Foster NE (2013). Conceptual overlap of psychological constructs in low back pain. Pain.

[41] Mittinty MM, Vanlint S, Stocks N, Mittinty MN, Moseley GL (2018). Exploring effect of pain education on chronic pain patients' expectation of recovery and pain intensity. Scand J Pain.

[42] Mansell G, Storheim K, Lochting I, Werner EL, Grotle M (2017). Identification of indirect effects in a cognitive patient education (COPE) intervention for low Back pain. Phys Ther.

[43] Hall AM, Kamper SJ, Emsley R, Maher CG (2016). Does pain-catastrophising mediate the effect of tai chi on treatment outcomes for people with low back pain?. Complement Thr Med.

[44] Smeets RJ, Vlaeyen JW, Kester AD, Knottnerus JA (2006). Reduction of pain catastrophizing mediates the outcome of both physical and cognitive-behavioral treatment in chronic low back pain. J Pain.

[45] Spinhoven P, Ter Kuile M, Kole-Snijders AM, Hutten Mansfeld M, Den Ouden DJ, Vlaeyen JW (2004). Catastrophizing and internal pain control as mediators of outcome in the multidisciplinary treatment of chronic low back pain. Eur J Pain.

[46] Lee H, Hubscher M, Moseley GL, Kamper SJ, Traeger AC, Mansell G, McAuley JH (2015). How does pain lead to disability? A systematic review and meta-analysis of mediation studies in people with back and neck pain. Pain.

[47] Jackson T, Wang Y, Wang Y, Fan H (2014). Self-efficacy and chronic pain outcomes: a meta-analytic review. J Pain.

[48] Baird AJ, Haslam RA (2013). Exploring differences in pain beliefs within and between a large nonclinical (workplace) population and a clinical (chronic low back pain) population using the pain beliefs questionnaire. Phys Ther.

[49] Walsh DA, Radcliffe JC (2002). Pain beliefs and perceived physical disability of patients with chronic low back pain. Pain.

[50] Linton SJ, Flink IK, Vlaeyen JWS (2018). Understanding the etiology of chronic pain from a psychological perspective. Phys Ther.

[51] van Dijk W, Faber MJ, Tanke MA, Jeurissen PP, Westert GP (2016). Medicalisation and Overdiagnosis: what society does to medicine. Int J Health Policy Manag.

[52] Bandura A (1977). Self-efficacy: toward a unifying theory of behavioral change. Psychol Rev.

[53] Fordyce WE (1976). Behavioural methods for chronic pain and illness.

[54] Bunzli S, Gillham D, Esterman A (2011). Physiotherapy-provided operant conditioning in the management of low back pain disability: a systematic review. Physiother Res Int.

[55] Laird RA, Gilbert J, Kent P, Keating JL (2014). Comparing lumbo-pelvic kinematics in people with and without back pain: a systematic review and meta-analysis. BMC Musculoskelet Disord.

[56] Hodges PW, Smeets RJ (2015). Interaction between pain, movement, and physical activity: short-term benefits, long-term consequences, and targets for treatment. Clin J Pain.

[57] Gizzi L, Rohrle O, Petzke F, Falla D. People with low back pain show reduced movement complexity during their most active daily tasks. Eur J Pain. 2018;[Epub ahead of print]. 10.1002/ejp.1318 30246275

[58] Sjogaard G, Sogaard K (2014). Muscle activity pattern dependent pain development and alleviation. J Electromyogr Kinesiol.

[59] Vlaeyen JW, Kole-Snijders AM, Boeren RG, van Eek H (1995). Fear of movement/(re)injury in chronic low back pain and its relation to behavioral performance. Pain.

[60] Crombez G, Vlaeyen JW, Heuts PH, Lysens R (1999). Pain-related fear is more disabling than pain itself: evidence on the role of pain-related fear in chronic back pain disability. Pain.

[61] Wideman TH, Adams H, Sullivan MJ (2009). A prospective sequential analysis of the fear-avoidance model of pain. Pain.

[62] Crombez G, Eccleston C, Van Damme S, Vlaeyen JW, Karoly P (2012). Fear-avoidance model of chronic pain: the next generation. Clin J Pain.

[63] Vlaeyen JW, Linton SJ (2012). Fear-avoidance model of chronic musculoskeletal pain: 12 years on. Pain.

[64] Du S, Hu L, Dong J, Xu G, Chen X, Jin S, Zhang H, Yin H (2017). Self-management program for chronic low back pain: a systematic review and meta-analysis. Patient Educ Couns.

[65] Mansell G, Hall A, Toomey E (2016). Behaviour change and self-management interventions in persistent low back pain. Best Pract Res Clin Rheumatol.

[66] Bodenheimer T, Lorig K, Holman H, Grumbach K (2002). Patient self-management of chronic disease in primary care. JAMA.

[67] Tougas ME, Hayden JA, McGrath PJ, Huguet A, Rozario S (2015). A systematic review exploring the social cognitive theory of self-regulation as a framework for chronic health condition interventions. PLoS One.

[68] Beck JS (2011). Cognitive behavior therapy, basics and beyond, 2nd edn.

[69] Wong JJ, Cote P, Sutton DA, Randhawa K, Yu H, Varatharajan S, Goldgrub R, Nordin M, Gross DP, Shearer HM (2017). Clinical practice guidelines for the noninvasive management of low back pain: a systematic review by the Ontario protocol for traffic injury management (OPTIMa) collaboration. Eur J Pain.

[70] Hall A, Richmond H, Copsey B, Hansen Z, Williamson E, Jones G, Fordham B, Cooper Z, Lamb S (2018). Physiotherapist-delivered cognitive-behavioural interventions are effective for low back pain, but can they be replicated in clinical practice? A systematic review. Disabil Rehabil.

[71] Clarke CL, Ryan CG, Martin DJ (2011). Pain neurophysiology education for the management of individuals with chronic low back pain: systematic review and meta-analysis. Man Ther.

[72] Brunner E, De Herdt A, Minguet P, Baldew SS, Probst M (2013). Can cognitive behavioural therapy based strategies be integrated into physiotherapy for the prevention of chronic low back pain? A systematic review. Disabil Rehabil.

[73] Ramond-Roquin A, Bouton C, Gobin-Tempereau AS, Airagnes G, Richard I, Roquelaure Y, Huez JF (2014). Interventions focusing on psychosocial risk factors for patients with non-chronic low back pain in primary care--a systematic review. Fam Pract.

[74] Richmond H, Hall AM, Copsey B, Hansen Z, Williamson E, Hoxey-Thomas N, Cooper Z, Lamb SE (2015). The effectiveness of cognitive Behavioural treatment for non-specific low Back pain: a systematic review and meta-analysis. PLoS One.

[75] Linton SJ (2000). A review of psychological risk factors in back and neck pain. Spine (Phila Pa 1976).

[76] Henschke N, Ostelo RW, van Tulder MW, Vlaeyen JW, Morley S, Assendelft WJ, Main CJ. Behavioural treatment for chronic low-back pain. Cochrane Database Syst Rev. 2010;2010(7):CD002014. 10.1002/14651858.CD002014.pub3 PMC7065591 20614428

[77] Baez S, Hoch MC, Hoch JM. Evaluation of cognitive behavioral interventions and psychoeducation implemented by rehabilitation specialists to treat fear-avoidance beliefs in patients with low Back pain: a systematic review. Arch Phys Med Rehabil. 2018;99(11):2287–98. 10.1016/j.apmr.2017.11.003 29247627

[78] Qaseem A, Wilt TJ, McLean RM, Forciea MA (2017). Clinical guidelines Committee of the American College of P: noninvasive treatments for acute, subacute, and chronic low Back pain: a clinical practice guideline from the American College of Physicians. Ann Intern Med.

[79] Maher C, Underwood M, Buchbinder R (2017). Non-specific low back pain. Lancet.

[80] Chou R, Deyo R, Friedly J, Skelly A, Hashimoto R, Weimer M, Fu R, Dana T, Kraegel P, Griffin J (2016). Noninvasive treatments for low Back pain. Comparative effectiveness review no 169.

[81] Stevens ML, Lin CC, de Carvalho FA, Phan K, Koes B, Maher CG (2017). Advice for acute low back pain: a comparison of what research supports and what guidelines recommend. Spine J.

[82] Melzack R (2005). Evolution of the neuromatrix theory of pain. The Prithvi raj lecture: presented at the third world congress of world Institute of Pain, Barcelona 2004. Pain practice : the official journal of World Institute of Pain.

[83] Linton SJ, Shaw WS (2011). Impact of psychological factors in the experience of pain. Phys Ther.

[84] Main CJ, Foster N, Buchbinder R (2010). How important are back pain beliefs and expectations for satisfactory recovery from back pain?. Best Pract Res Clin Rheumatol.

[85] Baird A, Sheffield D. The relationship between pain beliefs and physical and mental health outcome measures in chronic low back pain: direct and indirect effects. Healthcare (Basel). 2016;4(3):2–11. 10.3390/healthcare4030058 PMC5041059 27548244

[86] Hurley J, O'Keeffe M, O'Sullivan P, Ryan C, McCreesh K, O'Sullivan K (2016). Effect of education on non-specific neck and low back pain: a meta-analysis of randomized controlled trials. Man Ther.

[87] Traeger AC, Hubscher M, Henschke N, Moseley GL, Lee H, McAuley JH (2015). Effect of primary care-based education on reassurance in patients with acute low Back pain: systematic review and meta-analysis. JAMA Intern Med.

[88] Di Iorio A, Abate M, Guralnik JM, Bandinelli S, Cecchi F, Cherubini A, Corsonello A, Foschini N, Guglielmi M, Lauretani F (2007). From chronic low back pain to disability, a multifactorial mediated pathway: the InCHIANTI study. Spine (Phila Pa 1976).

[89] Hammill RR, Beazell JR, Hart JM (2008). Neuromuscular consequences of low back pain and core dysfunction. Clin Sports Med.

[90] Smeets RJ, Wittink H, Hidding A, Knottnerus JA (2006). Do patients with chronic low back pain have a lower level of aerobic fitness than healthy controls?: are pain, disability, fear of injury, working status, or level of leisure time activity associated with the difference in aerobic fitness level?. Spine (Phila Pa 1976).

[91] van Weering MG, Vollenbroek-Hutten MM, Tonis TM, Hermens HJ (2009). Daily physical activities in chronic lower back pain patients assessed with accelerometry. Eur J Pain.

[92] van den Berg-Emons RJ, Schasfoort FC, de Vos LA, Bussmann JB, Stam HJ (2007). Impact of chronic pain on everyday physical activity. Eur J Pain.

[93] Verbunt JA, Smeets RJ, Wittink HM (2010). Cause or effect? Deconditioning and chronic low back pain. Pain.

[94] Smith BE, Hendrick P, Bateman M, Moffatt F, Rathleff MS, Selfe J, Logan P, Smith TO (2017). Should exercises be painful in the management of chronic musculoskeletal pain? A systematic review and meta-analysis. Br J Sports Med.

[95] Steffens D, Maher CG, Pereira LS, Stevens ML, Oliveira VC, Chapple M, Teixeira-Salmela LF, Hancock MJ (2016). Prevention of low Back pain: a systematic review and meta-analysis. JAMA Intern Med.

[96] Saragiotto BT, Maher CG, Yamato TP, Costa LO, Menezes Costa LC, Ostelo RW, Macedo LG (2016). Motor control exercise for chronic non-specific low-back pain. Cochrane Database Syst Rev.

[97] Macedo LG, Saragiotto BT, Yamato TP, Costa LO, Menezes Costa LC, Ostelo RW, Maher CG (2016). Motor control exercise for acute non-specific low back pain. Cochrane Database Syst Rev.

[98] Gordon R, Bloxham S. A Systematic Review of the Effects of Exercise and Physical Activity on Non-Specific Chronic Low Back Pain. Healthcare (Basel). 2016;4(2):1–19. 10.3390/healthcare4020022 PMC4934575 27417610

[99] Searle A, Spink M, Ho A, Chuter V (2015). Exercise interventions for the treatment of chronic low back pain: a systematic review and meta-analysis of randomised controlled trials. Clin Rehabil.

[100] van Middelkoop M, Rubinstein SM, Verhagen AP, Ostelo RW, Koes BW, van Tulder MW (2010). Exercise therapy for chronic nonspecific low-back pain. Best Pract Res Clin Rheumatol.

[101] Kristensen J, Franklyn-Miller A (2012). Resistance training in musculoskeletal rehabilitation: a systematic review. Br J Sports Med.

[102] Miyamoto GC, Lin CC, Cabral CMN, van Dongen JM, van Tulder MW. Cost-effectiveness of exercise therapy in the treatment of non-specific neck pain and low back pain: a systematic review with meta-analysis. Br J Sports Med. 2018;[Epub ahead of print]. 10.1136/bjsports-2017-098765 29678893

[103] Choi BK, Verbeek JH, Tam WW, Jiang JY (2010). Exercises for prevention of recurrences of low-back pain. Cochrane Database Syst Rev.

[104] Bigos SJ, Holland J, Holland C, Webster JS, Battie M, Malmgren JA (2009). High-quality controlled trials on preventing episodes of back problems: systematic literature review in working-age adults. Spine J.

[105] Shiri R, Coggon D, Falah-Hassani K (2018). Exercise for the prevention of low Back pain: systematic review and meta-analysis of controlled trials. Am J Epidemiol.

[106] Slade SC, Keating JL (2006). Trunk-strengthening exercises for chronic low back pain: a systematic review. J Manip Physiol Ther.

[107] van Tulder M, Malmivaara A, Esmail R, Koes B (2000). Exercise therapy for low back pain: a systematic review within the framework of the cochrane collaboration back review group. Spine.

[108] Stuber KJ, Bruno P, Sajko S, Hayden JA (2014). Core stability exercises for low back pain in athletes: a systematic review of the literature. Clin J Sport Med.

[109] Vila-Cha C, Falla D, Farina D (2010). Motor unit behavior during submaximal contractions following six weeks of either endurance or strength training. J Appl Physiol (1985).

[110] McKenzie R, May S. The Lumbar Spine: Mechanical Diagnosis & Therapy 2, Vol Set (801–2), vol. 1 and 2. Waikanae: Spinal Publications; 2006.

[111] Hayden JA, van Tulder MW, Malmivaara A, Koes BW (2005). Exercise therapy for treatment of non-specific low back pain. Cochrane Database Syst Rev.

[112] Yamato TP, Maher CG, Saragiotto BT, Hancock MJ, Ostelo RW, Cabral CM, Menezes Costa LC, Costa LO (2015). Pilates for low back pain. Cochrane Database Syst Rev.

[113] Macedo LG, Maher CG, Latimer J, McAuley JH (2009). Motor control exercise for persistent, nonspecific low back pain: a systematic review. Phys Ther.

[114] Luomajoki HA, Bonet Beltran MB, Careddu S, Bauer CM (2018). Effectiveness of movement control exercise on patients with non-specific low back pain and movement control impairment: a systematic review and meta-analysis. Musculoskelet Sci Pract.

[115] Russo M, Deckers K, Eldabe S, Kiesel K, Gilligan C, Vieceli J, Crosby P (2018). Muscle control and non-specific chronic low Back pain. Neuromodulation.

[116] Garber CE, Blissmer B, Deschenes MR, Franklin BA, Lamonte MJ, Lee IM, Nieman DC, Swain DP, American College of Sports M (2011). American College of Sports Medicine position stand. Quantity and quality of exercise for developing and maintaining cardiorespiratory, musculoskeletal, and neuromotor fitness in apparently healthy adults: guidance for prescribing exercise. Med Sci Sports Exerc.

[117] O'Keeffe M, Purtill H, Kennedy N, Conneely M, Hurley J, O'Sullivan P, Dankaerts W, O'Sullivan K (2016). Comparative effectiveness of conservative interventions for nonspecific chronic spinal pain: physical, behavioral/psychologically informed, or combined? A systematic review and meta-analysis. J Pain.

[118] O'Sullivan P, Caneiro JP, O'Keeffe M, O'Sullivan K (2016). Unraveling the complexity of low Back pain. J Orthop Sports Phys Ther.

[119] Tousignant-Laflamme Y, Martel MO, Joshi AB, Cook CE (2017). Rehabilitation management of low back pain - it's time to pull it all together!. J Pain Res.

[120] Falla D, Hodges PW (2017). Individualized exercise interventions for spinal pain. Exerc Sport Sci Rev.

[121] O'Sullivan K, Dankaerts W, O'Sullivan L, O'Sullivan PB (2015). Cognitive functional therapy for disabling nonspecific chronic low Back pain: multiple case-cohort study. Phys Ther.

[122] Hill JC, Whitehurst DG, Lewis M, Bryan S, Dunn KM, Foster NE, Konstantinou K, Main CJ, Mason E, Somerville S (2011). Comparison of stratified primary care management for low Back pain with current best practice (STarT Back): a randomised controlled trial. Lancet.

[123] Vibe Fersum K, O'Sullivan P, Skouen JS, Smith A, Kvale A (2013). Efficacy of classification-based cognitive functional therapy in patients with non-specific chronic low back pain: a randomized controlled trial. Eur J Pain.

[124] Bovend'Eerdt TJ, Botell RE, Wade DT (2009). Writing SMART rehabilitation goals and achieving goal attainment scaling: a practical guide. Clin Rehabil.

[125] Lamb SE, Lall R, Hansen Z, Castelnuovo E, Withers EJ, Nichols V, Griffiths F, Potter R, Szczepura A, Underwood M (2010). A multicentred randomised controlled trial of a primary care-based cognitive behavioural programme for low Back pain. The Back skills training (BeST) trial. Health Technol Assess.

[126] Lamb SE, Hansen Z, Lall R, Castelnuovo E, Withers EJ, Nichols V, Potter R, Underwood MR (2010). Back skills training trial i: group cognitive behavioural treatment for low-back pain in primary care: a randomised controlled trial and cost-effectiveness analysis. Lancet.

[127] Gardner H (1999). Intelligence reframed: multiple intelligences for teh 21st century.

[128] Pashler H, McDaniel M, Rohrer D, Bjork R (2008). Learning styles: concepts and evidence. Psychol Sci Public Interest.

[129] Schmidt HG, Rotgans JI, Yew EH (2011). The process of problem-based learning: what works and why. Med Educ.

[130] Trigwell K (2001). Judging university teaching. Int J Acad Dev.

[131] Buccino G, Colage I, Gobbi N, Bonaccorso G (2016). Grounding meaning in experience: a broad perspective on embodied language. Neurosci Biobehav Rev.

[132] Gibbs RW (2003). Embodied experience and linguistic meaning. Brain Lang.

[133] Gauvin MG, Riddle DL, Rothstein JM (1990). Reliability of clinical measurements of forward bending using the modified fingertip-to-floor method. Phys Ther.

[134] Ito T, Shirado O, Suzuki H, Takahashi M, Kaneda K, Strax TE (1996). Lumbar trunk muscle endurance testing: an inexpensive alternative to a machine for evaluation. Arch Phys Med Rehabil.

[135] Arab AM, Salavati M, Ebrahimi I, Ebrahim Mousavi M (2007). Sensitivity, specificity and predictive value of the clinical trunk muscle endurance tests in low back pain. Clin Rehabil.

[136] Moreland J, Finch E, Stratford P, Balsor B, Gill C (1997). Interrater reliability of six tests of trunk muscle function and endurance. J Orthop Sports Phys Ther.

[137] Strand LI, Anderson B, Lygren H, Skouen JS, Ostelo R, Magnussen LH (2011). Responsiveness to change of 10 physical tests used for patients with back pain. Phys Ther.

[138] Andersson EI, Lin CC, Smeets RJ (2010). Performance tests in people with chronic low back pain: responsiveness and minimal clinically important change. Spine (Phila Pa 1976).

[139] Aasa B, Berglund L, Michaelson P, Aasa U (2015). Individualized low-load motor control exercises and education versus a high-load lifting exercise and education to improve activity, pain intensity, and physical performance in patients with low back pain: a randomized controlled trial. J Orthop Sports Phys Ther.

[140] van Erp RMA, Huijnen IPJ, Koke AJA, Abbink FE, den Hollander M, Smeets R (2017). Development and content of the biopsychosocial primary care intervention ‘Back on Track’ for a subgroup of people with chronic low back pain. Physiotherapy.

[141] Hurley DA, Murphy LC, Hayes D, Hall AM, Toomey E, McDonough SM, Lonsdale C, Walsh NE, Guerin S, Matthews J (2016). Using intervention mapping to develop a theory-driven, group-based complex intervention to support self-management of osteoarthritis and low back pain (SOLAS). Implement Sci.

[142] Hurley DA, Hall AM, Currie-Murphy L, Pincus T, Kamper S, Maher C, McDonough SM, Lonsdale C, Walsh NE, Guerin S (2016). Theory-driven group-based complex intervention to support self-management of osteoarthritis and low back pain in primary care physiotherapy: protocol for a cluster randomised controlled feasibility trial (SOLAS). BMJ Open.

[143] van Erp RM, Huijnen IP, Verbunt JA, Smeets RJ (2015). A biopsychosocial primary care intervention (Back on track) versus primary care as usual in a subgroup of people with chronic low back pain: protocol for a randomised, controlled trial. J Physiother.

[144] Keogh A, Matthews J, Segurado R, Hurley DA (2018). Feasibility of training physical therapists to deliver the theory-based self-Management of Osteoarthritis and low Back Pain through Activity and skills (SOLAS) intervention within a trial. Phys Ther.

[145] Banerjee A, Hendrick P, Bhattacharjee P, Blake H (2018). A systematic review of outcome measures utilised to assess self-management in clinical trials in patients with chronic pain. Patient Educ Couns.

[146] Leysen M, Nijs J, Meeus M, Paul van Wilgen C, Struyf F, Vermandel A, Kuppens K, Roussel NA (2015). Clinimetric properties of illness perception questionnaire revised (IPQ-R) and brief illness perception questionnaire (brief IPQ) in patients with musculoskeletal disorders: a systematic review. Man Ther.

[147] Broadbent E, Wilkes C, Koschwanez H, Weinman J, Norton S, Petrie KJ (2015). A systematic review and meta-analysis of the brief illness perception questionnaire. Psychol Health.

[148] Waddell G, Newton M, Henderson I, Somerville D, Main CJ (1993). A fear-avoidance beliefs questionnaire (FABQ) and the role of fear-avoidance beliefs in chronic low back pain and disability. Pain.

[149] Grotle M, Brox JI, Vollestad NK (2006). Reliability, validity and responsiveness of the fear-avoidance beliefs questionnaire: methodological aspects of the Norwegian version. J Rehabil Med.

[150] Stroyer J, Essendrop M, Jensen LD, Warming S, Avlund K, Schibye B (2007). Validity and reliability of self-assessed physical fitness using visual analogue scales. Percept Mot Skills.

[151] Denteneer L, Stassijns G, De Hertogh W, Truijen S, Van Daele U (2017). Inter- and Intrarater reliability of clinical tests associated with functional lumbar segmental instability and motor control impairment in patients with low Back pain: a systematic review. Arch Phys Med Rehabil.

[152] Jones CJ, Rikli RE, Beam WC (1999). A 30-s chair-stand test as a measure of lower body strength in community-residing older adults. Res Q Exerc Sport.

[153] Primdahl J, Wagner L, Horslev-Petersen K (2010). Self-efficacy in rheumatoid arthritis: translation and test of validity, reliability and sensitivity of the Danish version of the rheumatoid arthritis self-efficacy questionnaire (RASE). Musculoskeletal Care.

[154] Lauridsen HH, Hartvigsen J, Manniche C, Korsholm L, Grunnet-Nilsson N (2006). Danish version of the Oswestry disability index for patients with low back pain. Part 2: sensitivity, specificity and clinically significant improvement in two low back pain populations. Eur Spine J.

[155] Lauridsen HH, Hartvigsen J, Manniche C, Korsholm L, Grunnet-Nilsson N (2006). Danish version of the Oswestry disability index for patients with low back pain. Part 1: cross-cultural adaptation, reliability and validity in two different populations. Eur Spine J.

[156] Woby SR, Watson PJ, Roach NK, Urmston M (2004). Are changes in fear-avoidance beliefs, catastrophizing, and appraisals of control, predictive of changes in chronic low back pain and disability?. Eur J Pain.

[157] Strong J, Ashton R, Chant D (1991). Pain intensity measurement in chronic low back pain. Clin J Pain.

[158] Andersen JS, Olivarius Nde F, Krasnik A (2011). The Danish National Health Service Register. Scand J Public Health.

[159] Stapelfeldt CM, Jensen C, Andersen NT, Fleten N, Nielsen CV (2012). Validation of sick leave measures: self-reported sick leave and sickness benefit data from a Danish national register compared to multiple workplace-registered sick leave spells in a Danish municipality. BMC Public Health.

