# Supplementary material for: GLA:D® Back group-based patient education integrated with exercises to support self-management of back pain - development, theories and scientific evidence -
Source: BMC Musculoskelet Disord. 2018 Nov 29;19:418. doi: 10.1186/s12891-018-2334-x (PMC6267880; doi:10.1186/s12891-018-2334-x)
Supplement: Supplementary file 1 — GLA:D® Back Exercise program. The individual depicted in the images provided her written informed consent for the publication of these identifiable images (GLA:D® is a registered Trademark of the University of Southern Denmark: The name can only be used for an intervention if all criteria described by the University of Southern Denmark are met). (PDF 650 kb) [file 12891_2018_2334_MOESM1_ESM.pdf]

GLA:D® Back

WARM UP

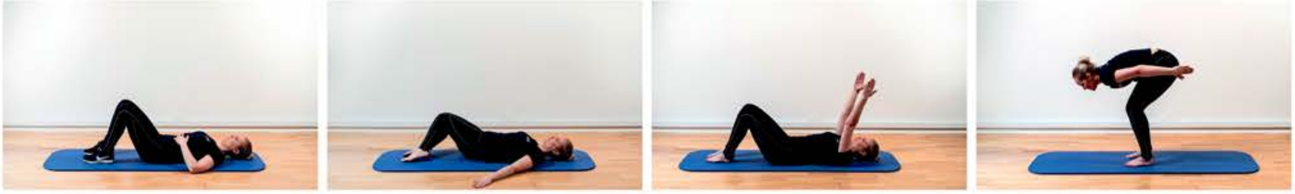

BACK EXTENSORS

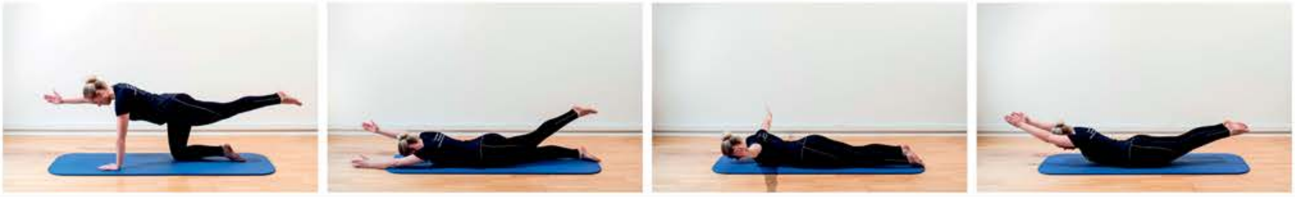

ABDOMINALS

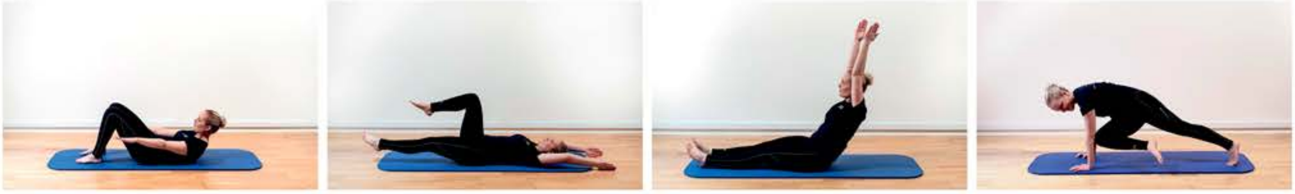

LATERAL GLUTEALS

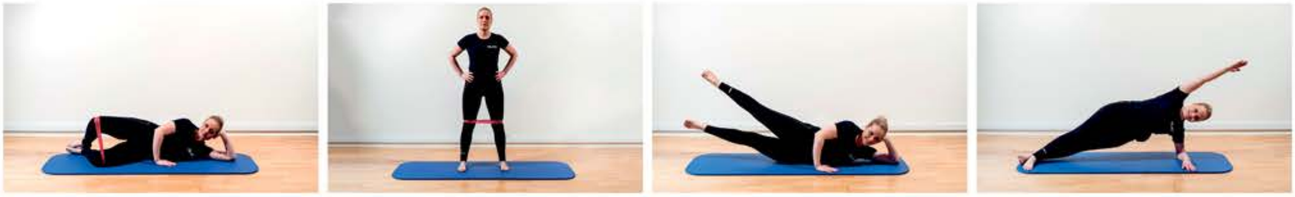

BACK ROTATION

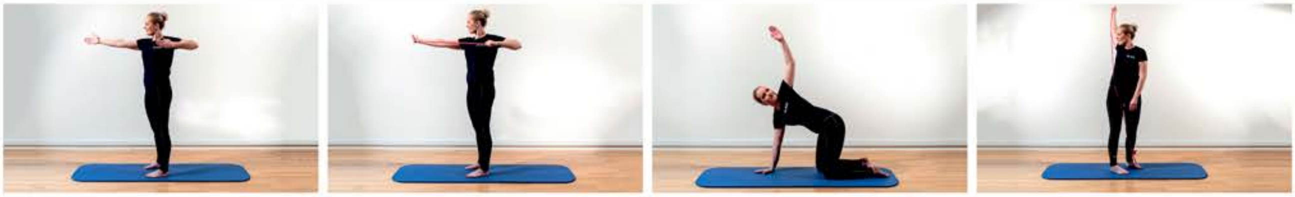

POSTERIOR GLUTEALS

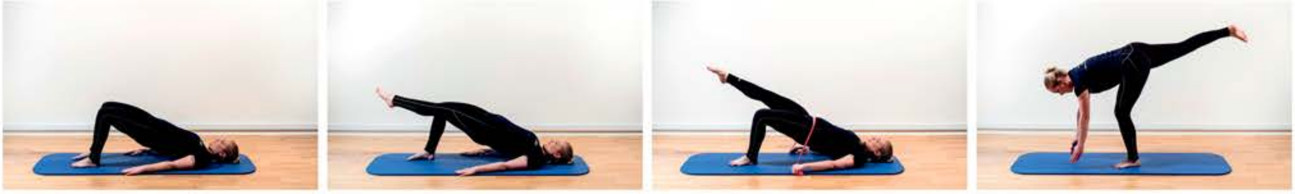

LEG MUSCLES

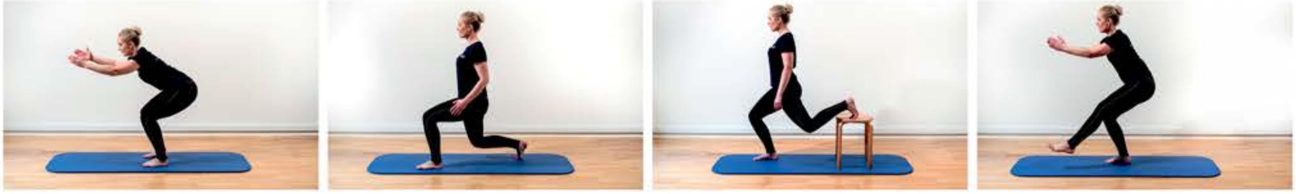

OBLIQUE ABOMINALS

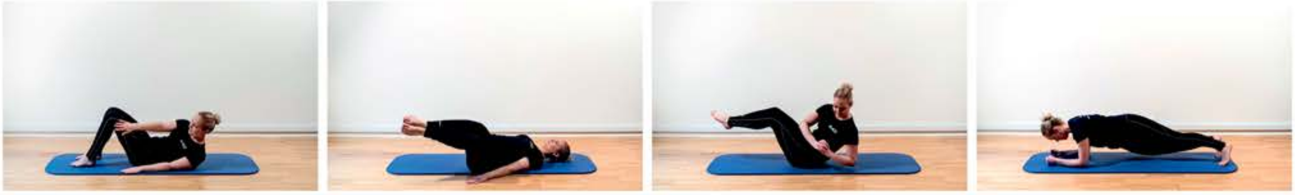

FLEXIBILITY

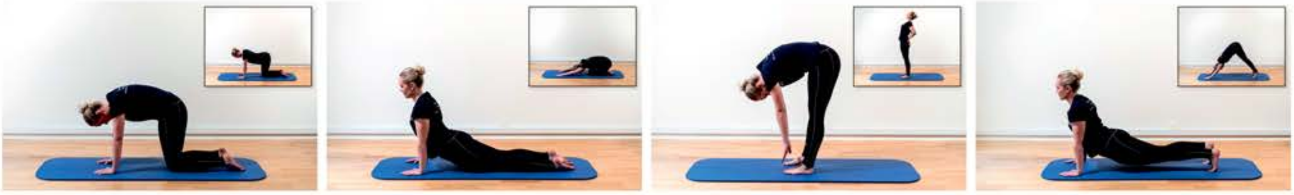

PRINT: PRINT & SIGN, SDU

SDU
