# Supplementary material for: GLA:D® Back group-based patient education integrated with exercises to support self-management of back pain - development, theories and scientific evidence -
Source: BMC Musculoskelet Disord. 2018 Nov 29;19:418. doi: 10.1186/s12891-018-2334-x (PMC6267880; doi:10.1186/s12891-018-2334-x)
Supplement: Supplementary file 2 — Suggestions for different types of instruction for the exercises. (DOCX 42 kb) [file 12891_2018_2334_MOESM2_ESM.docx]

# Supplementary File 1

## Suggestions for different types of instruction for the exercises

### Primary goal of instructions:

- the patient feels competent and safe in performing different movements
- the patient feels the exercises are positive (emotionally, physically, and cognitively) and links movement with positive thoughts
- the patient develops skills to respond to pain during movement
- the patient does not focus on structural changes as a cause of pain
- the patient takes ownership of his/her own training

### Safety

- the patient is not instructed in the one ‘correct’ way to perform the exercises
- the concepts of ‘stability’ and ‘instability’ are not mentioned.
- the patient is introduced to a repertoire of ways to move, achieving trust and confidence in back movements by trying out different ways of doing the exercises that allow the brain to connect motion to experiences other than pain.

#### Suggested messages / instructions

Create confidence and ownership by avoiding correcting errors: instead, guide in exploring other starting positions and movements so that the patient finds his/her way to do the exercises. For example:

- "You can try how it works if you change the movement, for example lift the pelvis more / less"
- "See if the movement feels different if you do it slower / faster?"
- "Does it make a difference if you tighten the muscles in the buttocks/belly?"
- "Feel how the muscles of your abdomen, legs and back work”
- "Think of how wonderful it is that you are working to strengthen your back, where every exercise is a step in the right direction"
- "Give yourself time to complete the entire range of movement"

### Enjoy movement

- The patient is guided to enjoy finding his/her way of doing the exercises, not to focus on the pain, limitations, or the "correct" way of doing the exercises
- The patient is guided to positive experiences with movements of the body and back, experiences in improving function of the back with training

#### Suggested messages / instructions

- "How can you do this exercise, so that it feels really good - experiment with pace and movement." I will tell you if you do something really wrong "
- "Compliment yourself on being here and for doing something"
- "Remember to enjoy the sense of doing something good for yourself"

### Handling of pain provocation

- The patient avoids having a focus on pain; the aim is to avoid the patient registering pain as a signal of injury, so that increasing pain sensitisation is neglected, and an automatic response to pain no longer results in the ending of an activity
- the patient explores movement as a means to overcoming pain. The patient works with acceptance of pain, adapts gradually to stress, and learns to distinguish between limitations of the back and fear of movement

#### Suggested messages / instructions

Create awareness that feelings of distress are unhelpful when related to pain. Include examples from pain education such as sensitisation, the alarm system, “a song on the brain”

- "You're doing the exercise just fine. If you start to feel pain, this may be your alarm system turning on, so then you check that the inputs coming from your sensitive area are ok "
- "Your pain alarm has started and would like you to stop. Try to see what happens if you ignore it by continuing the activity a little longer - as if your body will continue to work just fine "
- "Do you think that it may be your brain that has started singing the usual song? Can you distract yourself from it? "
- "Try and see what happens if you focus on something else, some good thoughts or your breathing"
- "Try to move in a relaxed way - possibly by changing the pace a little "
- "Your brain connects movement with pain and cheats you out of a good experience. Try to [make suggestions for changes] to get some new experiences. Your brain likes re-programming! "

### Causes of pain

- The patient no longer believes that his/her back problems cannot be improved due to structural damage or abnormality
- the patient learns that the range of normal standards for the back is extensive; that a number of conditions can make the back more sensitive, but that does not prevent recovery through training; that focus is on improving function and pain management, rather than eliminating the back problem

#### Suggested messages / instructions

Underline that it is not possible to determine exactly which structure is causing pain when the patient points out that it may be his/her disc, facet joint or certain muscles that hurt.

- "It is normal to have a scoliosis or changes in the structures of the back; many have it without pain"
- "Try not to think about what's wrong with your back when you feel pain. You risk activating the alarm system even more, just because it gets attention. You may want to help yourself by counting backwards from 100, planning a good dinner, re-living your best vacation / work / experiences with your children, telling a joke .... "
- "Your back is strong. Your joints can carry you all day, it's not causing you any harm by doing these exercises"
- "Pain comes and goes, often not in a recognisable pattern. When you wonder about the causes, your brain pays too much attention to your back. Instead, is there anything that helps you? Is there anything else you can do?”
- "We cannot change the imbalances and degenerative changes you have, but they are not an obstacle to making you feel better"

### Ownership of exercises

- The patient should avoid relying on others or therapists to handle his/her back problems
- The patient feels competent to manage his/her training and exercises now and into the future

#### Suggested messages / instructions

Avoid correcting the exercises, but give the patient instructions on how to explore and adjust the exercises

- "Try to work with different speeds, ranges of movements, and relaxation in other muscles. If it feels better, do it "
- "You now know different levels of this exercise - so you can customise them to your current capacity"
- "I'm sure you're in control of it"
- "You are an expert in your own training, you know what works well for you"
- "I will guide you so that you are able to be your own coach"
- "When an exercise does not work well, we can try different variations until you've found what feels good"
- "Ask if you're in doubt that you might be doing something bad for your back"
- “Find your favourite exercise, which can be helpful on a bad back day”

# Appendix 2

# Literature for exercises

1. Aagaard P: **Training-induced changes in neural function**. *Exerc Sport Sci Rev* 2003, **31**(2):61-67.

2. Aagaard P, Andersen JL, Dyhre-Poulsen P, Leffers AM, Wagner A, Magnusson SP, Halkjaer-Kristensen J, Simonsen EB: **A mechanism for increased contractile strength of human pennate muscle in response to strength training: changes in muscle architecture**. *J Physiol* 2001, **534**(Pt. 2):613-623.

3. McGill S: **Core Training: Evidence Translating to Better Performance and Injury Prevention**. *Strength & Conditioning Journal* 2010, **32**(3):33-46.

4. Kristensen J, Franklyn-Miller A: **Resistance training in musculoskeletal rehabilitation: a systematic review**. *Br J Sports Med* 2012, **46**(10):719-726.

5. Slade SC, Keating JL: **Trunk-strengthening exercises for chronic low back pain: a systematic review**. *J Manipulative Physiol Ther* 2006, **29**(2):163-173.

6. van Tulder M, Malmivaara A, Esmail R, Koes B: **Exercise therapy for low back pain: A systematic review within the framework of the cochrane collaboration back review group**. *Spine* 2000, **25**(21):2784-2796.

7. Stuber KJ, Bruno P, Sajko S, Hayden JA: **Core stability exercises for low back pain in athletes: a systematic review of the literature**. *Clin J Sport Med* 2014, **24**(6):448-456.

8. Vila-Cha C, Falla D, Farina D: **Motor unit behavior during submaximal contractions following six weeks of either endurance or strength training**. *J Appl Physiol (1985)* 2010, **109**(5):1455-1466.

9. Yamato TP, Maher CG, Saragiotto BT, Hancock MJ, Ostelo RW, Cabral CM, Menezes Costa LC, Costa LO: **Pilates for low back pain**. *Cochrane Database Syst Rev* 2015(7):CD010265.

10. Richardson C, Jull G, Hodges P, Hides J: **Therapeutic Exercise for Spinal Segmental Stabilization in Low Back Pain**. London: Churchill Livingstone 1999.

11. Sahrmann SA: **Diagnosis and Treatment of Movement Impairment Syndromes**. St. Louis: Mosby; 2002.

12. O'Sullivan P: **Diagnosis and classification of chronic low back pain disorders: maladaptive movement and motor control impairments as underlying mechanism**. *Man Ther* 2005, **10**(4):242-255.

13. Saragiotto BT, Maher CG, Yamato TP, Costa LO, Menezes Costa LC, Ostelo RW, Macedo LG: **Motor control exercise for chronic non-specific low-back pain**. *Cochrane Database Syst Rev* 2016, **1**(1):CD012004.

14. Macedo LG, Saragiotto BT, Yamato TP, Costa LO, Menezes Costa LC, Ostelo RW, Maher CG: **Motor control exercise for acute non-specific low back pain**. *Cochrane Database Syst Rev* 2016, **2**:CD012085.

15. Searle A, Spink M, Ho A, Chuter V: **Exercise interventions for the treatment of chronic low back pain: a systematic review and meta-analysis of randomised controlled trials**. *Clin Rehabil* 2015, **29**(12):1155-1167.

16. Macedo LG, Maher CG, Latimer J, McAuley JH: **Motor control exercise for persistent, nonspecific low back pain: a systematic review**. *Phys Ther* 2009, **89**(1):9-25.

17. Luomajoki HA, Bonet Beltran MB, Careddu S, Bauer CM: **Effectiveness of movement control exercise on patients with non-specific low back pain and movement control impairment: A systematic review and meta-analysis**. *Musculoskelet Sci Pract* 2018, **36**:1-11.

18. McKenzie R, May S: **The Lumbar Spine: Mechanical Diagnosis & Therapy 2, Vol Set (801-2)**, vol. 1 and 2: Spinal Publications; 2006.

19. van Middelkoop M, Rubinstein SM, Verhagen AP, Ostelo RW, Koes BW, van Tulder MW: **Exercise therapy for chronic nonspecific low-back pain**. *Best Pract Res Clin Rheumatol* 2010, **24**(2):193-204.

20. Hayden JA, van Tulder MW, Malmivaara A, Koes BW: **Exercise therapy for treatment of non-specific low back pain**. *Cochrane Database Syst Rev* 2005(3):CD000335.

21. Gomes-Neto M, Lopes JM, Conceicao CS, Araujo A, Brasileiro A, Sousa C, Carvalho VO, Arcanjo FL: **Stabilization exercise compared to general exercises or manual therapy for the management of low back pain: A systematic review and meta-analysis**. *Phys Ther Sport* 2017, **23**:136-142.

22. Steffens D, Maher CG, Pereira LS, Stevens ML, Oliveira VC, Chapple M, Teixeira-Salmela LF, Hancock MJ: **Prevention of Low Back Pain: A Systematic Review and Meta-analysis**. *JAMA Intern Med* 2016, **176**(2):199-208.

23. Falla D, Hodges PW: **Individualized Exercise Interventions for Spinal Pain**. *Exerc Sport Sci Rev* 2017, **45**(2):105-115.

24. Hodges PW, Smeets RJ: **Interaction between pain, movement, and physical activity: short-term benefits, long-term consequences, and targets for treatment**. *Clin J Pain* 2015, **31**(2):97-107.

25. Choi BK, Verbeek JH, Tam WW, Jiang JY: **Exercises for prevention of recurrences of low-back pain**. *Cochrane Database Syst Rev* 2010(1):CD006555.

26. Bigos SJ, Holland J, Holland C, Webster JS, Battie M, Malmgren JA: **High-quality controlled trials on preventing episodes of back problems: systematic literature review in working-age adults**. *Spine J* 2009, **9**(2):147-168.

27. Shiri R, Coggon D, Falah-Hassani K: **Exercise for the Prevention of Low Back Pain: Systematic Review and Meta-Analysis of Controlled Trials**. *Am J Epidemiol* 2018, **187**(5):1093-1101.

28. O'Keeffe M, Hayes A, McCreesh K, Purtill H, O'Sullivan K: **Are group-based and individual physiotherapy exercise programmes equally effective for musculoskeletal conditions? A systematic review and meta-analysis**. *Br J Sports Med* 2017, **51**(2):126-132.

29. van Middelkoop M, Rubinstein SM, Kuijpers T, Verhagen AP, Ostelo R, Koes BW, van Tulder MW: **A systematic review on the effectiveness of physical and rehabilitation interventions for chronic non-specific low back pain**. *Eur Spine J* 2011, **20**(1):19-39.

30. Garber CE, Blissmer B, Deschenes MR, Franklin BA, Lamonte MJ, Lee IM, Nieman DC, Swain DP, American College of Sports M: **American College of Sports Medicine position stand. Quantity and quality of exercise for developing and maintaining cardiorespiratory, musculoskeletal, and neuromotor fitness in apparently healthy adults: guidance for prescribing exercise**. *Med Sci Sports Exerc* 2011, **43**(7):1334-1359.
